# Identification of DSB-1, a Protein Required for Initiation of Meiotic Recombination in *Caenorhabditis elegans*, Illuminates a Crossover Assurance Checkpoint

**DOI:** 10.1371/journal.pgen.1003679

**Published:** 2013-08-08

**Authors:** Ericca L. Stamper, Stacia E. Rodenbusch, Simona Rosu, Julie Ahringer, Anne M. Villeneuve, Abby F. Dernburg

**Affiliations:** 1 Department of Molecular and Cell Biology and California Institute for Quantitative Biosciences (QB3), University of California, Berkeley; Berkeley, California, United States of America; 2 Department of Developmental Biology and Genetics, Stanford University School of Medicine, Stanford, California, United States of America; 3 The Gurdon Institute and Department of Genetics, University of Cambridge, Cambridge, United Kingdom; 4 Department of Genome Dynamics, Life Sciences Division, Lawrence Berkeley National Laboratory, Berkeley, California, United States of America; 5 Howard Hughes Medical Institute, Chevy Chase, Maryland, United States of America; The University of North Carolina at Chapel Hill, United States of America

## Abstract

Meiotic recombination, an essential aspect of sexual reproduction, is initiated by programmed DNA double-strand breaks (DSBs). DSBs are catalyzed by the widely-conserved Spo11 enzyme; however, the activity of Spo11 is regulated by additional factors that are poorly conserved through evolution. To expand our understanding of meiotic regulation, we have characterized a novel gene, *dsb-1*, that is specifically required for meiotic DSB formation in the nematode *Caenorhabditis elegans*. DSB-1 localizes to chromosomes during early meiotic prophase, coincident with the timing of DSB formation. DSB-1 also promotes normal protein levels and chromosome localization of DSB-2, a paralogous protein that plays a related role in initiating recombination. Mutations that disrupt crossover formation result in prolonged DSB-1 association with chromosomes, suggesting that nuclei may remain in a DSB-permissive state. Extended DSB-1 localization is seen even in mutants with defects in early recombination steps, including *spo-11*, suggesting that the absence of crossover precursors triggers the extension. Strikingly, failure to form a crossover precursor on a single chromosome pair is sufficient to extend the localization of DSB-1 on all chromosomes in the same nucleus. Based on these observations we propose a model for crossover assurance that acts through DSB-1 to maintain a DSB-permissive state until all chromosome pairs acquire crossover precursors. This work identifies a novel component of the DSB machinery in *C. elegans*, and sheds light on an important pathway that regulates DSB formation for crossover assurance.

## Author Summary

For most eukaryotes, recombination between homologous chromosomes during meiosis is an essential aspect of sexual reproduction. Meiotic recombination is initiated by programmed double-strand breaks in DNA, which have the potential to induce mutations if not efficiently repaired. To better understand the mechanisms that govern the initiation of recombination and regulate the formation of double-strand breaks, we use the nematode *Caenorhabditis elegans* as a model system. Here we describe a new gene, *dsb-1*, that is required for double-strand break formation in *C. elegans*. Through analysis of the encoded DSB-1 protein we illuminate an important regulatory pathway that promotes crossover recombination events on all chromosome pairs to ensure successful meiosis.

## Introduction

Formation of crossovers between homologous chromosomes is essential for successful execution of the meiotic program in most sexually reproducing organisms. In addition to shuffling genetic information between parental chromosomes, crossovers, together with cohesion between sister chromatids, create physical links between homologous chromosomes that enable their segregation to daughter cells during the first meiotic division [1], [2]. Disruption of crossover formation leads to chromosome nondisjunction and the formation of aneuploid gametes, and thereby greatly reduces fertility.

Meiotic recombination is initiated by programmed DNA double-strand breaks (DSBs), a subset of which is repaired to form crossovers between homologous chromosomes (for a review, see [3]). While a minimum number of DSBs is needed to promote the necessary crossovers on all chromosome pairs, excessive DSBs could threaten genome integrity. The number of meiotic DSBs in various organisms appears to be highly regulated, presumably to balance the crossover requirement with the risk of mutation. The timing of programmed DSBs during early meiotic prophase is also tightly controlled to maximize the likelihood of productive repair [4], [5]. For example, in *Saccharomyces cerevisiae*, the activities of cell cycle-regulated kinases involved in DNA replication ensure that that DSBs occur only after DNA synthesis is complete [6]–[10]. DSB formation must also be inactivated during meiotic prophase to allow for repair prior to the meiotic divisions. Mechanisms that terminate DSB formation are not well understood, although recent studies have shown that the ATM/ATR family of DNA damage response kinases is involved in down-regulating the number of DSBs in mice, *S. cerevisiae*, and *Drosophila melanogaster* [11]–[14]. Further investigations are needed to better understand the mechanisms underlying these various aspects of DSB regulation.

Meiotic DSBs are catalyzed by the widely conserved, topoisomerase-related enzyme Spo11 [15], [16]. Although Spo11 is essential for DSB formation, it does not function alone. In various organisms – including fungi, plants, and animals – additional proteins required for meiotic DSBs have been identified (for a review, see [17]). Unlike Spo11, other known factors involved in DSB formation are poorly conserved. For example, of five meiosis-specific DSB proteins found in *S. cerevisiae*, only two (Rec114 and Mei4) have known orthologs in other phyla; and even these two proteins are absent in several species, including *Caenorhabditis elegans*, *D. melanogaster*, and *Neurospora crassa* [18]. Additional DSB proteins have also been identified in other organisms, but none are ubiquitous among eukaryotes [5], .

The nematode *C. elegans* has emerged as a valuable model system for molecular analysis of meiosis. As in other eukaryotes, SPO-11 catalyzes the formation of meiotic DSBs [23]. MRE-11 and RAD-50 are also required for DSB formation [24], [25] as in *S. cerevisiae* [17], but these proteins have other essential roles in DNA metabolism, including in the resection of meiotic DSBs [3], [26]. In *C. elegans*, as in other species, meiosis-specific chromosome architecture contributes to DSB proficiency. In particular, in the absence of HTP-3, an integral component of chromosome axes, DSBs are abolished or sharply reduced [27]. The related protein HTP-1, which is also associated with the axial elements, may also contribute to DSB formation, while other axial components appear to be dispensable for DSBs [28]–[30]. Roles for axis components homologous to HTP-3 and HTP-1 in promoting DSBs have also been demonstrated in other organisms [3], [31], [32]. Additionally, the meiotic kinase CHK-2, which regulates many key events during early meiotic prophase, is required for programmed DSBs in *C. elegans* [33]. Several other factors are known to influence meiotic DSB formation, but their effects may be indirect. These include the chromatin-associated proteins HIM-5, HIM-17, and XND-1, which promote normal levels of meiotic DSBs, but whose functions are pleiotropic and not well understood 34–36. Apart from SPO-11, no protein that specifically functions in initiating recombination has previously been reported. Some aspects of *C. elegans* meiosis are unusual among model organisms, including the fact that synapsis between homologous chromosomes is independent of recombination [23]. Thus, analysis of DSB regulation in *C. elegans* will likely reveal both conserved aspects of meiosis and how regulatory circuits are remodeled during evolution.

Here we identify a novel gene, *dsb-1* (*double-strand break factor 1*), that is required for meiotic DSB formation in *C. elegans*. *dsb-1* mutants lack meiotic DSBs, and show meiotic defects similar to *spo-11* mutants. DSB-1 localizes to meiotic chromosomes coincident with the time of DSB formation, in a manner dependent on the CHK-2 kinase. We also find that a variety of mutations that disrupt crossover formation on one or more chromosomes extend the chromosomal localization of DSB-1, suggesting that the DSB-permissive state may be prolonged. Based on these observations, we infer the existence of a regulatory circuit in which meiotic nuclei monitor the recombination status of each chromosome pair and act through DSB-1 to maintain a DSB-permissive state until all chromosome pairs have attained crossover-competent recombination intermediates.

## Results

### Identification of *dsb-1*, a New Gene Required for Meiotic DSB Formation

**Figure 1:**
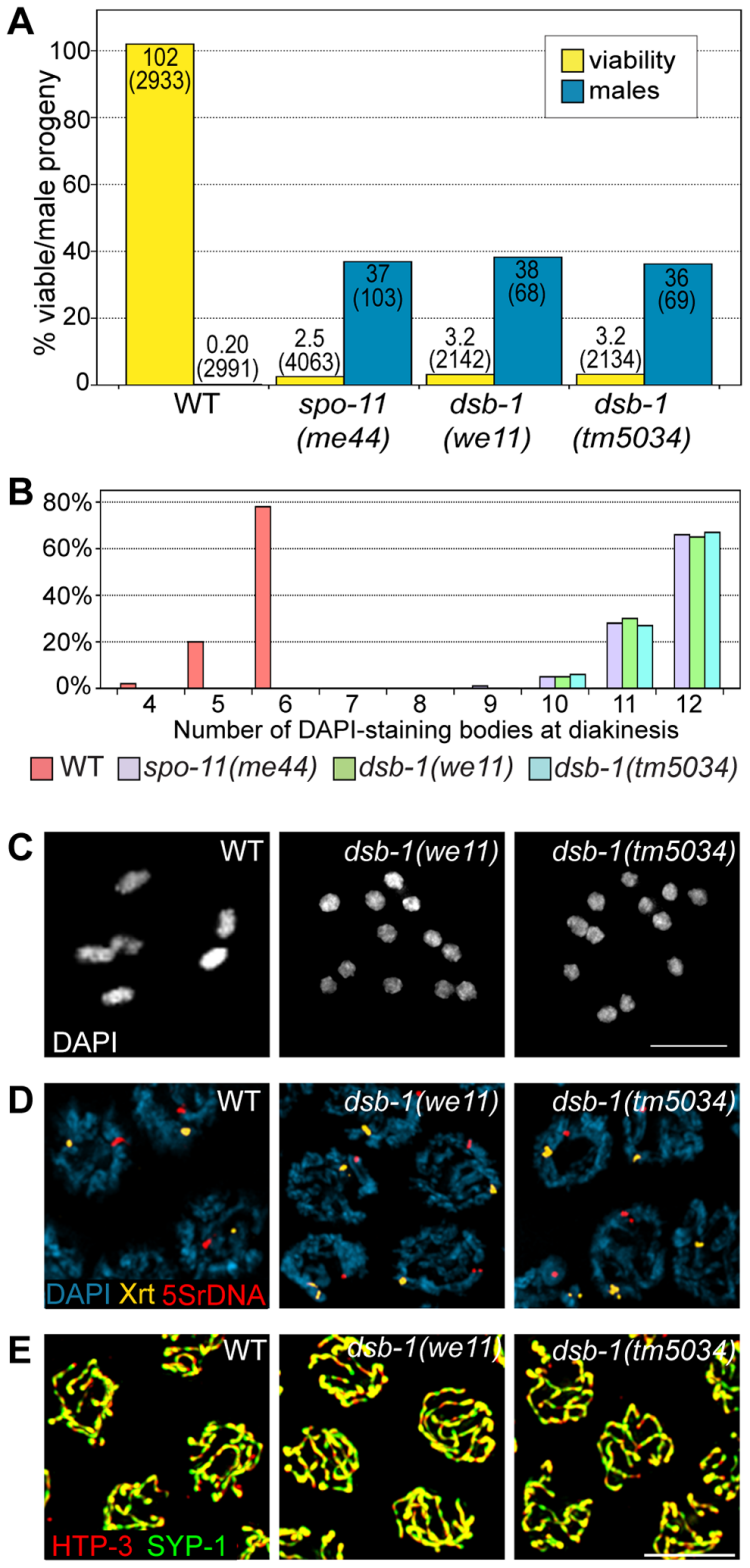
*dsb-1* mutants lack meiotic crossovers but are proficient for homologous chromosome pairing and synapsis. (A) Quantification of viable and male self-progeny for the indicated genotypes is shown. Homozygous *dsb-1(we11* and *tm5034)* hermaphrodites produce many inviable and male self-progeny compared to wild-type (WT) animals, similar to *spo-11* hermaphrodites. For each bar, the upper number indicates the percentage, and the lower number in parentheses indicates the total number of fertilized eggs (for viability) or adult progeny (for males) counted. (B) Histogram showing the number of DAPI-staining bodies observed in oocytes at diakinesis for the indicated genotypes. 100 oocytes were counted for each genotype. (C) Each panel shows a representative DAPI-stained oocyte nucleus at diakinesis for the indicated genotype. WT oocytes display 6 DAPI-staining bodies (bivalents), while *dsb-1* oocytes display 12 DAPI-staining bodies (univalents). (D) Fluorescence *in situ* hybridization against the 5S rDNA genomic locus (5SrDNA) and a locus on the right end of the X chromosome (Xrt) indicated that pairing is not disrupted in *dsb-1* mutants. (E) Synapsis was assessed by immunofluorescence staining of the axial element protein HTP-3 and central region protein SYP-1, components of the synaptonemal complex. Full colocalization of the two markers indicates fully synapsed chromosomes. Scale bars, 5 µm.

In *C. elegans*, mutations that impair meiotic chromosome segregation result in embryonic lethality and a high incidence of males (*XO*) among the surviving progeny [37]. The *dsb-1(we11)* mutant was isolated in a genetic screen for maternal-effect embryonic lethality, and was found to produce a high fraction of males among its few surviving self-progeny. A targeted deletion allele of the affected gene, *dsb-1(tm5034)*, was generated independently (see below), and results in defects identical to *dsb-1(we11)* based on all assays described here. Whereas self-fertilizing wild-type hermaphrodites produce nearly 100% viable progeny and 0.2% males (Figure 1A, [37]), only 3% of progeny from self-fertilizing *dsb-1* mutant hermaphrodites survived to adulthood (n>2000; 12 broods), (Figure 1A, Table 1). Among these survivors, 36–38% were male (Figure 1A, Table 1). The brood size (number of fertilized eggs) of self-fertilizing *dsb-1* hermaphrodites was also reduced relative to wild-type animals (Table 1).

**Table 1:** Progeny viability, incidence of males, and brood size from *dsb-1* and *dsb-2* mutants.

Genotype	% Viability (n)	% Males (n)	Brood Size (n)
wild-type	102 (2933)	0.20 (2991)	285 (10)
*spo-11(me44)*	2.5 (4063)	37 (103)	343 (11)
*dsb-1(we11)*	3.2 (2142)	38 (68)	184 (12)
*dsb-1(tm5034)*	3.2 (2134)	36 (69)	178 (12)
*rol-1*	98 (1741)	0.06 (1705)	290 (6)
*rol-1 dsb-2(me96)*	29 (789)	15 (229)	263 (3)
*dsb-1(tm5034)*	3.7 (782)	38 (29)	196 (4)
*rol-1 dsb-2(me96); dsb-1(tm5034)*	3.8 (1500)	35 (57)	214 (7)

Quantification of the viable and male self-progeny from hermaphrodites of indicated genotypes, as well as average brood size (fertilized eggs), is shown. The numbers in parentheses indicate: fertilized eggs (for viability), adult progeny (for male counts), and broods (for brood size) counted. Each of the two sets of experiments was performed in parallel under identical conditions.

Chromosome segregation errors in meiosis often reflect defects in crossover formation between homologs. The levels of embryonic lethality and male progeny observed in *dsb-1* mutants are quantitatively similar to several previously characterized mutants that fail to make any crossovers during meiotic prophase, such as *spo-11* (Figure 1A, Table 1), *msh-5*, and *cosa-1* [23], [38], [39], suggesting that *dsb-1* mutants might also lack crossovers. Visualization of DAPI-stained oocytes at diakinesis provides a simple assay for crossover formation in *C. elegans*. In wild-type hermaphrodites, 6 DAPI-staining bodies are observed in each oocyte nucleus (average = 5.8, Figure 1B and 1C), corresponding to 6 pairs of homologous chromosomes, each held together by a chiasma [40]. In mutants that fail to make crossovers, oocytes typically display 12 DAPI-staining bodies. The number and morphology of DAPI-staining bodies observed in *dsb-1* mutant oocytes was similar to *spo-11* mutants (average = 11.6, Figure 1B and 1C), indicating an absence of chiasmata in *dsb-1* animals.

We investigated whether the disruption of crossover formation in *dsb-1* mutants might reflect a defect in homologous chromosome pairing or synapsis. Pairing was assessed using fluorescence *in situ* hybridization (Figure 1D). Early pachytene nuclei of both wild-type and *dsb-1* animals contained a single focus or closely apposed pair of foci, indicating that homologous chromosomes were paired (Figure 1D). Further, co-staining of the axial element protein HTP-3 and the synaptonemal complex central region protein SYP-1 indicated that chromosomes were fully synapsed by early pachytene in *dsb-1* animals (Figure 1E), as in wild-type animals. These results indicate that *dsb-1* mutants are proficient for homologous chromosome pairing and synapsis.

**Figure 2:**
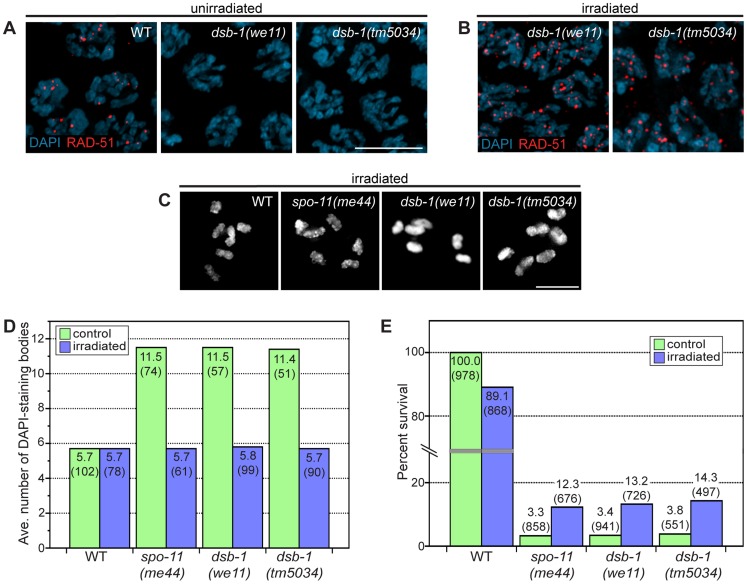
*dsb-1* is required to initiate meiotic recombination. (A, B) Immunofluorescence staining of RAD-51 was used as a cytological marker of early recombination intermediates in early pachytene nuclei in (A) untreated and (B) irradiated animals. Scale bar, 5 µm. (A) WT nuclei display abundant RAD-51; however, *dsb-1* mutants lack RAD-51 foci. (B) *dsb-1* mutants were gamma-irradiated (10 Gy) as young adults, and then assessed 2 hours later for the presence of RAD-51 foci. RAD-51 foci were present on chromosomes in *dsb-1* mutants after irradiation. (C, D) WT, *spo-11(me44)*, and *dsb-1* mutants were gamma-irradiated (10 Gy) as young adults. After 18 hours, irradiated and control animals were fixed, and the number of DAPI-staining bodies in oocytes at diakinesis was quantified. (C) Each panel shows DAPI-stained chromosomes in a single, representative oocyte at diakinesis for the indicated genotype. Similar to *spo-11* and WT, irradiated *dsb-1* mutants displayed 6 DAPI-staining bodies. Scale bar, 5 µm. (D) Quantification of the number of DAPI-staining bodies observed for each genotype in untreated and irradiated animals. The top number for each bar indicates the average number of DAPI-staining bodies and the number in parentheses indicates the number of ooctyes counted. (E) WT, *spo-11*, and *dsb-1* mutants were gamma-irradiated (10 Gy) at 4–5 hours post L4. Eggs laid 20–30 hours post irradiation were counted for progeny survival. The top number for each bar indicates the percent survival; the numbers in parentheses indicates the total number of eggs counted. As for *spo-11*, irradiation of *dsb-1* mutants partially rescued progeny survival.

To assess whether *dsb-1* mutants initiate meiotic recombination, we used antibodies against the DNA strand-exchange protein RAD-51, which binds to single-stranded regions adjacent to resected DSBs [26], [41], as a cytological marker of recombination intermediates [42], [43]. Whereas wild-type oocytes in early pachytene showed abundant RAD-51 foci, *dsb-1* gonads lacked RAD-51 staining (Figure 2A), indicating either failure to form DSBs or failure to load RAD-51. However, the lack of fragmented chromosomes at diakinesis seemed more consistent with an absence of DSBs.

To verify that *dsb-1* mutants are defective in DSB formation, and to rule out the possibility of defects in the loading of RAD-51 or downstream steps of the recombination pathway, we tested whether exogenous DSBs could rescue the recombination defects observed in *dsb-1* mutants. The same approach established a role for Spo11/SPO-11 in DSB formation [23], [44]. Young adult *dsb-1* mutant hermaphrodites were exposed to 10 Gy of gamma rays, a dose that has previously been shown to efficiently rescue crossovers in *spo-11* mutants with minimal associated lethality [25]. Wild-type and *spo-11* controls were performed in parallel. At appropriate times after irradiation the animals were assessed for RAD-51 foci, chiasmata, and progeny viability. At 2 hours post irradiation, *dsb-1* animals displayed abundant RAD-51 foci (Figure 2B), indicating that the mutants are proficient for resection and RAD-51 loading. At 18 hours post irradiation, both *spo-11* and *dsb-1* oocytes showed ∼6 DAPI-staining bodies (Figure 2C and 2D). Additionally, the viability of embryos laid 20–30 hours post irradiation increased significantly for both *spo-11* and *dsb-1* animals, but decreased slightly for wild-type, compared to unirradiated controls (Figure 2E). The ability of exogenous DSBs to rescue the recombination defects of *dsb-1* animals indicates that these mutants are specifically defective in meiotic DSB formation.

The defects observed in *dsb-1* mutant hermaphrodites are virtually indistinguishable from *spo-11(me44)* mutants, except that mutations in *dsb-1* were associated with reduced brood size (Table 1). Although *dsb-1(we11)* showed linkage to the middle of Chromosome IV, close to the *spo-11* locus, complementation tests revealed that *we11* is not an allele of *spo-11*. Quantitative RT-PCR also indicated that *spo-11* mRNA levels were unaffected in *dsb-1(we11)* mutants (Figure S1).

### *dsb-1* Encodes a Member of a Novel Protein Family

**Figure 3:**
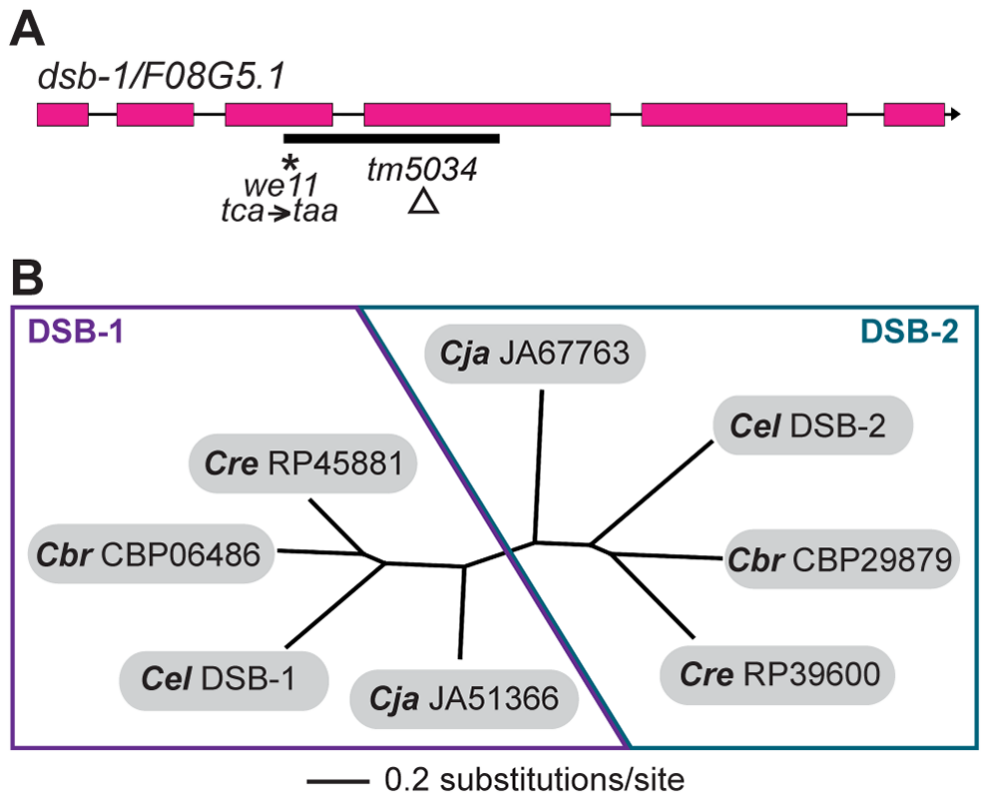
*dsb-1* is a novel gene that belongs to a poorly conserved gene family. (A) Structure of the *dsb-1* gene (*F08G5.1*) indicating the 2 mutant alleles analyzed in this study: *we11* and *tm5034*. The *we11* allele introduces a premature stop at codon 97, while the *tm5034* deletion allele causes a frameshift that introduces one amino acid followed by a stop codon after lysine 96. (B) Phylogenetic tree of DSB-1 homologs in *C. elegans*, *C. briggsae*, *C. remanei*, and *C. japonica*. Each species shown contains two paralogs belonging to DSB-1 protein family. These proteins appear to fall into 2 paralogous groups: the DSB-1 group and the DSB-2 group.

Whole-genome sequencing of backcrossed *dsb-1(we11)* animals identified several mutations in annotated coding sequences, including a nonsense mutation in the previously uncharacterized gene *F08G5.1* (Figure 3A), which encodes a predicted protein of 385 amino acids and seemed a plausible candidate based on its meiosis-enriched expression pattern [45]. We found that knockdown of *F08G5.1* expression via transgene-mediated cosuppression [46] caused embryonic lethality and male progeny, as well as strong reduction of chiasmata, in the oocytes of treated animals (data not shown), supporting the hypothesis that the *we11* mutation affects this gene. *we11* introduces a premature stop (*tac* = >*taa*) after lysine 96 (Figure 3A). A targeted deletion allele *(tm5034)* removes 290 bp from predicted exons 3 and 4 and the intervening intron (Figure 3A), resulting in a frameshift mutation that introduces a glutamine immediately followed by a stop codon after lysine 96. The phenotype of *dsb-1(tm5034)* mutants is indistinguishable from *dsb-1(we11)* (Figure 1 and 2, Table 1). Both are predicted to lack functional protein based on the early stop codons, and this conclusion is supported by immunofluorescence and immunoblotting experiments (below). Based on the evidence described above that mutations disrupting *F08G5.1* specifically interfere with meiotic double-strand break formation, we designated *F08G5.1* as *dsb-1*, for double-strand break factor 1.

The DSB-1 protein has no apparent homologs outside of the genus *Caenorhabditis*, including other nematode genera. Interestingly, the genomes of *C. elegans* and several other Caenorhabditids each contain 2 predicted paralogs. In an accompanying paper, Rosu *et al.* show that *dsb-1* paralog *F26H11.6/dsb-2* is also involved in meiotic DSB formation in *C. elegans* [47]. DSB-1, DSB-2, and their homologs cluster into two paralogous groups (Figure 3B). Even within *Caenorhabditis*, members of this protein family are not well conserved (Figure S2).

DSB-1 lacks identifiable domains that might give clues about its function in DSB formation. One notable feature is its high serine content: 60 of 385 amino acids (16%) are serine residues, compared to an average serine content of 8% encoded by all *C. elegans* ORFs [48]. Protein structure prediction algorithms indicate that each end of DSB-1 may form alpha-helix secondary structures, but the central portion of the protein, which is especially serine-rich, is predicted to be largely unstructured. This central region is also the least conserved portion of the protein (Figure S2). Five serine residues within the central region are followed by glutamine (Q), making them candidate phosphorylation targets for ATM or ATR DNA damage kinases. These clustered ATM/ATR consensus motifs are shared by other DSB-1 homologs, including DSB-2.

### DSB-1 Localizes to Chromosomes during Early Meiotic Prophase

**Figure 4:**
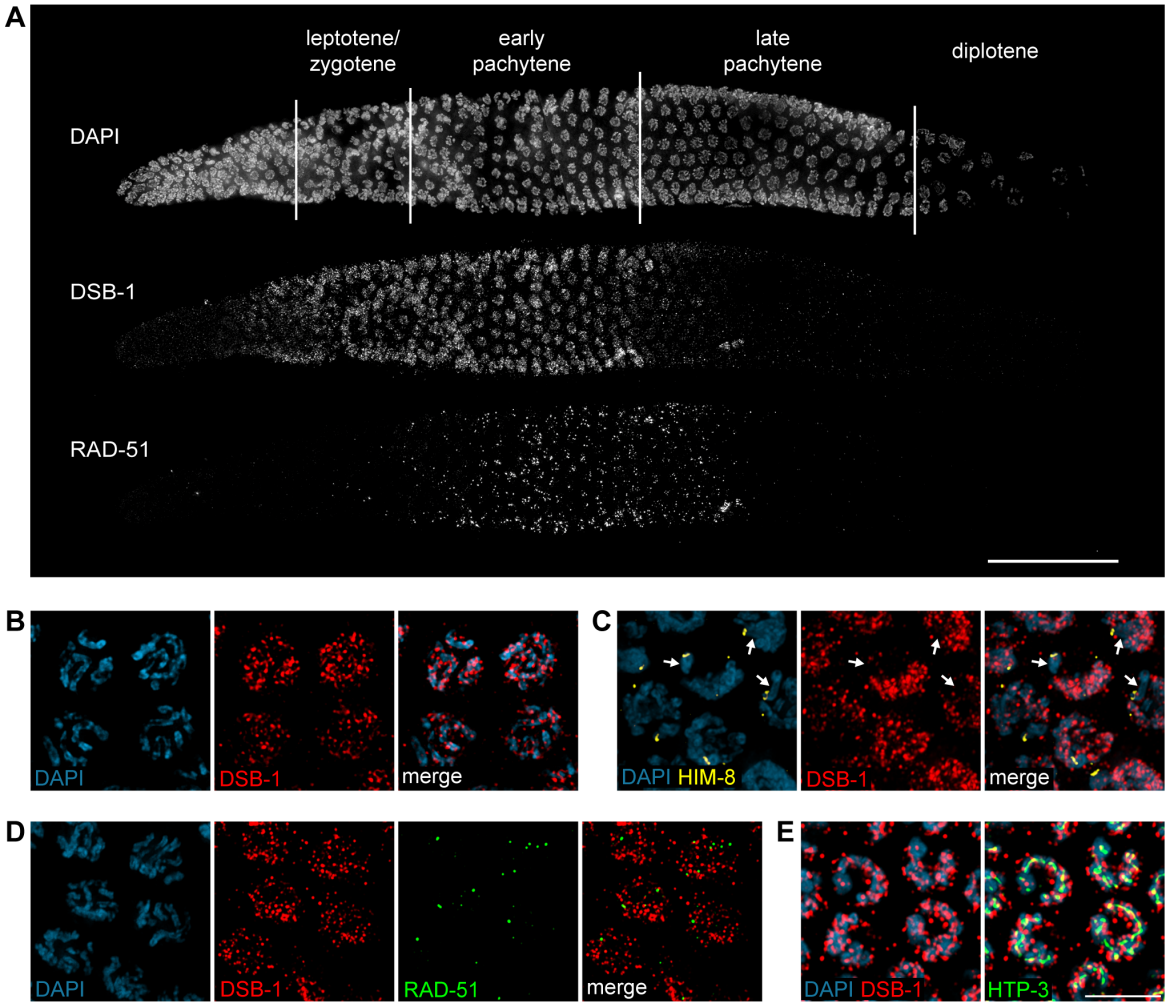
DSB-1 localizes to chromosomes during early meiotic prophase. (A) Composite projection image of a gonad from a WT animal showing DAPI and immunofluorescence staining for DSB-1 and RAD-51. Scale bar, 30 µm. (B) Partial projection image of pachytene meiotic nuclei from WT showing DAPI and immunofluorescence staining of DSB-1. Scale bar, 5 µm. (C) Immunofluorescence staining of DSB-1 and HIM-8 in early pachytene nuclei in WT. HIM-8 was used to mark the X chromosome (arrows). DSB-1 staining was reduced on the X chromosome. (D) Partial projection image of early pachytene nuclei in WT showing immunofluorescence staining of RAD-51 and DSB-1. Little colocalization was observed between DSB-1 and RAD-51. (E) Partial projection image of early pachytene nuclei from WT showing immunofluorescence staining of DSB-1, HTP-3, and DAPI. Only a subset of DSB-1 colocalized with chromosome axes, marked by HTP-3. Scale bar, 5 µm.

To further probe the role of DSB-1 in the formation of meiotic DSBs, we generated an antibody against the full-length protein expressed in *E. coli*. Immunofluorescence staining revealed that DSB-1 is absent from somatic nuclei, and specifically localizes to chromosomes during early meiotic prophase (Figure 4A and 4B), while *dsb-1* mutants showed only background staining (Figure S3). Accumulation of DSB-1 on chromosomes was first observed in nuclei marked by crescent-shaped DAPI-staining morphology, corresponding to the “transition zone” (leptotene/zygotene), and disappeared at mid-pachytene (Figure 4A). Chromosomal localization of DSB-1 preceded the appearance of RAD-51 foci, consistent with an early role for DSB-1 in meiotic recombination (Figure 4A). Thus, the localization of DSB-1 to chromosomes corresponds to the period during which DSBs are likely to be generated.

While most nuclei in the late pachytene region of the germline lacked DSB-1 staining, we consistently observed dispersed nuclei in this region that retained bright fluorescence (Figure 4A and S4). These nuclei also contained abundant RAD-51 foci and frequently displayed compact chromosome morphology resembling that seen in the transition zone, along with evidence of asynapsed chromosomes (Figure S4A and S4B). We tested whether these late DSB-1 positive nuclei might be apoptotic by examining *ced-4* mutants, which lack germline apoptosis 49,50, and found that they were still present in similar numbers (data not shown). The persistence of RAD-51 foci and asynapsed chromosomes suggest that these nuclei may be delayed in completing synapsis or other prerequisites for crossover formation, a conclusion reinforced by further analysis of DSB-1 regulation, described below.

DSB-1 was distributed as a network of foci and stretches of staining on meiotic chromosomes (Figure 4B). One chromosome consistently showed weaker DSB-1 staining. This seemed likely to be the X chromosome, which has many unique features in the germline, including distinct chromatin marks 51,52 and genetic requirements for meiotic DSBs 35,36. Co-staining with antibodies against HIM-8, which specifically mark the X chromosome 53, confirmed that the chromosome pair with weaker DSB-1 staining was the X (Figure 4C).

DSB-1 and RAD-51 both localized to chromosomes during early pachytene (Figure 4A). However, we found that RAD-51 did not colocalize with DSB-1 (Figure 4D). Similar findings have been reported for DSB proteins in mice and *Schizosaccharomyces pombe* 18,54. This could indicate that DSB-1 does not act directly at DSB sites, or that it is removed from DSB sites prior to RAD-51 loading.

Meiotic chromosomes are believed to be organized as chromatin loops tethered at their bases to the proteinaceous chromosome axis 55,56. Based on work from *S. cerevisiae*, it has been proposed that DSBs occur at sites within chromatin loops that are recruited to the chromosome axis 57–59. Most DSB-1 staining was associated with the periphery of DAPI-stained chromosomes rather than axes (Figure 4E), suggesting that DSB-1 is primarily associated with chromatin loops. This localization pattern is similar to what has been observed for several DSB proteins in *S. cerevisiae* 6,60–63.

### Chromosome Localization Of Dsb-1 Is Independent Of Spo-11, The Mrn Complex, And A Properly Assembled Meiotic Chromosome Axis

**Figure 5:**
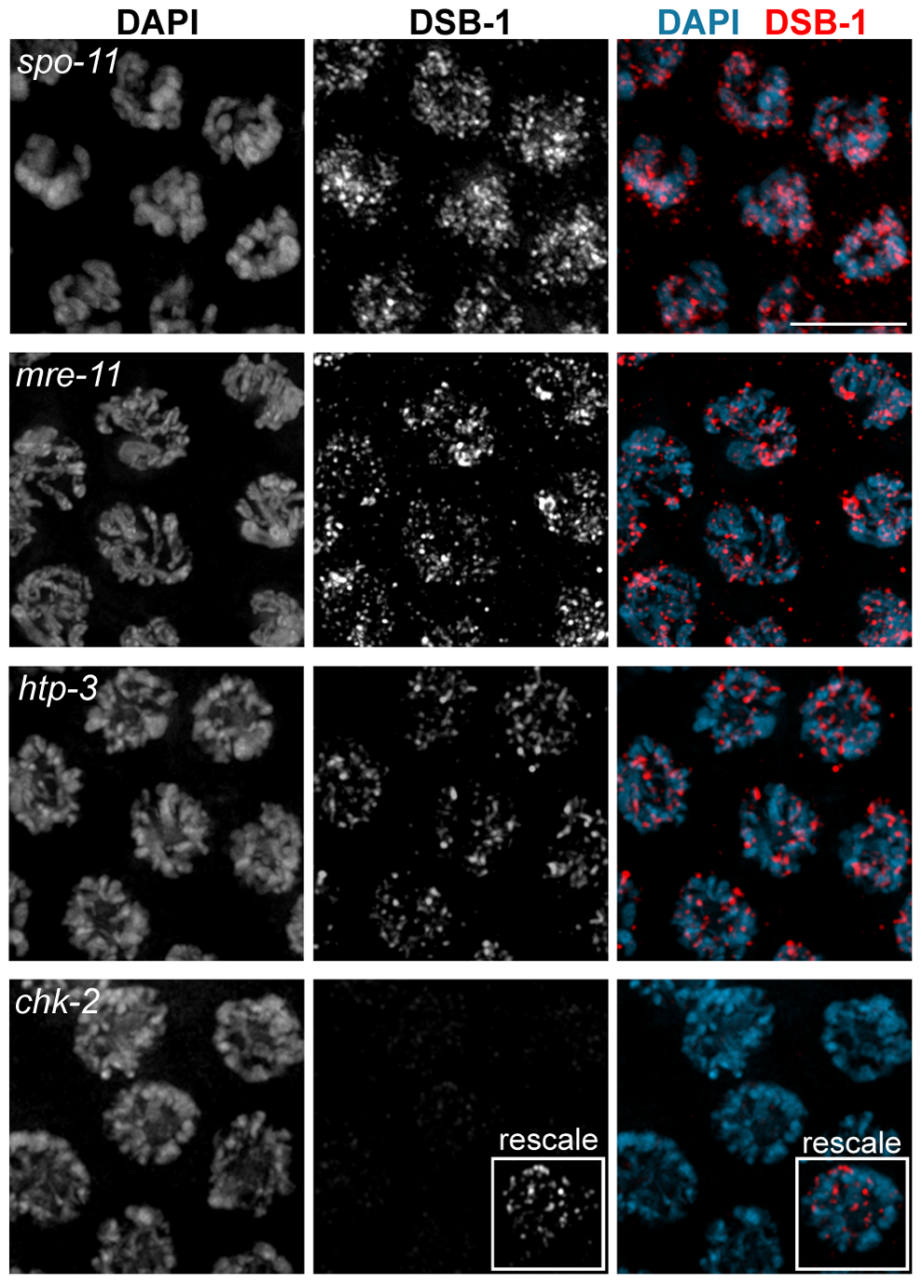
DSB-1 localization is independent of *spo-11*, *mre-11*, and *htp-3*, but requires *chk-2*. Immunofluorescence staining of DSB-1 with DAPI in pachytene nuclei from *spo-11(ok79)*, *mre-11*, *htp-3*, and *chk-2* mutant hermaphrodites. Localization of DSB-1 was not reduced in *spo-11* or *mre-11*, but was slightly reduced in *htp-3* mutants. Localization of DSB-1 in *chk-2* mutants was greatly reduced compared to WT, but was not abolished, as shown in inset, in which the DSB-1 signal has been rescaled to highlight the faint chromosomal staining. Scale bar, 5 µm.

We tested whether DSB-1 localization depends on other factors required for DSB formation. DSB-1 localized to meiotic chromosomes in the catalytically dead *spo-11(me44)* mutant 25, as well as in *spo-11(ok79)* mutants, which lack functional protein 23, indicating that DSB-1 localizes to chromosomes independently of DSBs and SPO-11 (Figure 5). DSB-1 localization was also independent of MRE-11 and RAD-50 (Figure 5, data not shown), which are required for DSB formation in *C. elegans* 24,25. In *htp-3* mutants, which lack an essential axial element component that is important for DSB formation 27, DSB-1 was detected on meiotic chromosomes (Figure 5), but the staining appeared somewhat reduced compared to wild-type nuclei.

### Chromosome Localization Of Dsb-1 Is Dependent On The Meiotic Kinase Chk-2

The CHK-2 kinase is essential for several key events during early meiotic prophase in *C. elegans*, including DSB formation and homolog pairing 33. We found that nuclear staining of DSB-1 was strongly reduced, albeit still detectable, in *chk-2(me64)* mutants (Figure 5). Although the intensity of DSB-1 staining was sharply reduced in *chk-2* mutants, it appears that faint fluorescence observed upon prolonged exposure reflects DSB-1, because the nuclear staining pattern resembles that seen in wild-type animals, and because chromosomal staining is not detected prior to meiotic entry. Western blot analysis revealed that DSB-1 protein is expressed in *chk-2* mutants, although the protein levels appear somewhat reduced compared to wild-type (data not shown). However, the reduction in DSB-1 protein levels in *chk-2* mutants does not appear to fully account for the sharply diminished chromosomal localization of DSB-1. These data indicate that DSB-1 localization to chromosomes is largely dependent on the CHK-2 kinase, and suggest that DSB-1 may act downstream of CHK-2 to promote DSBs.

### Disruption Of Crossover Formation Prolongs Chromosome Localization Of Dsb-1

**Figure 6:**
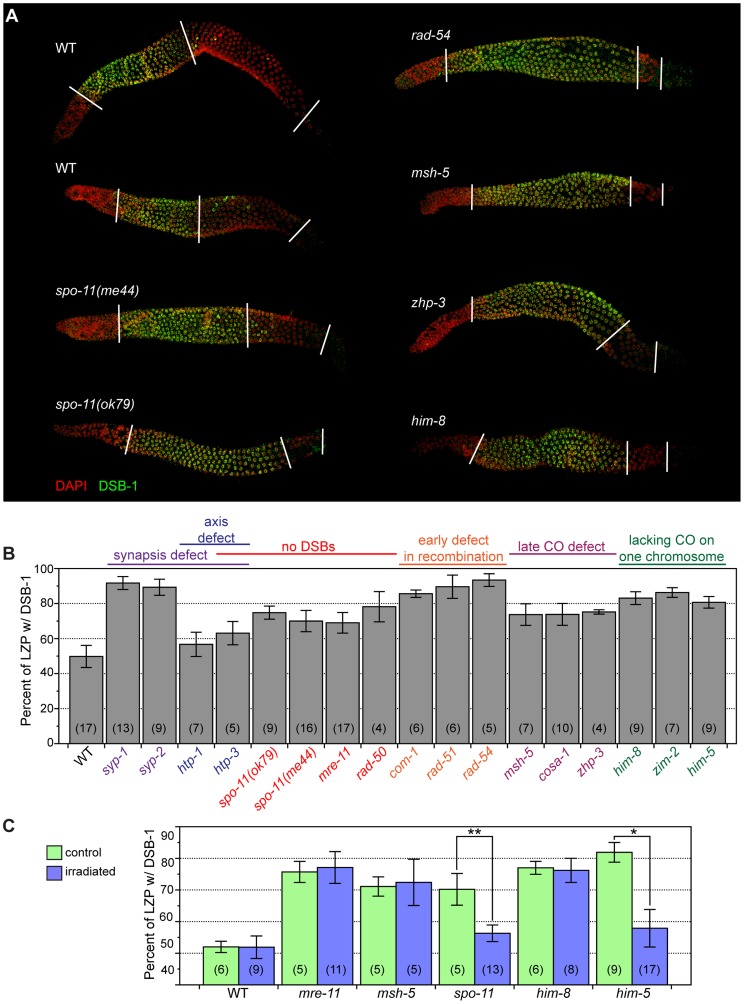
The region of the germline with nuclear DSB-1 localization is extended in mutants with impaired crossover formation. (A) Composite projection images of gonads from indicated genotypes showing immunofluorescence staining of DSB-1 and DAPI. Lines represent the start (left line) and end (right line) of the leptotene-zygotene-pachytene (LZP) region of the gonad, and the end of the zone of DSB-1 localization (middle line). (B) Quantification of the zone of DSB-1 localization, showing the percent, by length, of the LZP region positive for DSB-1 staining. Numbers in parentheses indicate the number of gonads quantified for each genotype. All genotypes showed significant differences from wild type, p<0.003 except for *htp-1*, for which p<0.05. Error bars indicate standard deviation. Genotypes are color-coded based on the category of meiotic defect that they cause (listed above the graph). (C) Quantification of the zone of DSB-1 localization in gamma-irradiated animals and unirradiated controls. Animals were irradiated (10 Gy) at 16 hours post L4, then dissected and fixed 8 hours later to measure the length of the zone of DSB-1 localization relative to the length of the LZP region. Numbers in parentheses indicate the number of gonad arms quantified. Error bars indicate standard deviation. **p = 0.0005, *p = 0.002.

In testing the genetic requirements for DSB-1 localization, we noticed that the zone of DSB-1 staining in the gonad was extended in mutants that disrupt crossover formation. Previous studies have reported a persistence of RAD-51 foci in numerous mutants that are proficient for DSBs but not for crossovers 43,64,65. In wild-type animals, and also in most mutants with extended RAD-51 staining, DSB-1 staining disappeared concomitant with, or slightly before, the disappearance of RAD-51 foci (Figure 4A and S5). Two exceptions were *rec-8* and *rad-54* mutants, in which DSB-1 staining disappeared by late pachytene, but RAD-51 staining persisted into diplotene (Figure 6, data not shown). Since DSB-1 is required for DSBs and its localization correlates with the timing of DSB formation, its presence on chromosomes may be indicative of proficiency for DSB formation. Although the presence of DSB-1 on chromosomes may not be sufficient for prolonged DSB formation, we interpret extension of the region of DSB-1 staining as evidence of a prolonged DSB-permissive state (see Discussion).

We quantified the extension of DSB-1 localization by comparing the length of the zone of DSB-1-positive nuclei to the total length of the region spanned by the transition zone through late pachytene nuclei, just before oocyte nuclei begin to form a single row near the bend region of the gonad, which coincides with diplotene (Figure 6A). We designated this entire zone as the LZP region (leptotene-zygotene-pachytene), although it also includes a few diplotene nuclei. We found that this metric – the length ratio of the DSB-1-positive region to the LZP region – was consistent across age-matched animals of the same genotype. In wild-type adult hermaphrodites, DSB-1 positive nuclei comprised about 50% of the length the LZP region (Figure 6A and 6B). However, in most mutants that disrupt crossover formation on one or more chromosomes, this zone of DSB-1 staining was significantly extended (Figure 6A and 6B). We saw some variability in this extension, which tended to correlate with the nature of the mutation: Mutations affecting late steps in the crossover pathway, including *msh-5(me23)* 38, *cosa-1(me13)* 39, *and zhp-3(jf61)* 66, extended the DSB-1 zone to ∼75%, of the LZP region (Figure 6A and 6B). Mutations that block earlier steps in homologous recombination, including *com-1(t1626)* 67, *rad-51(Ig8701)* 42, and *rad-54(tm1268)* 65, extended the DSB-1 zone even further, to ∼90% of the LZP region. Mutations that block crossover formation by disrupting synapsis, including *syp-1(me17)* 68 and *syp-2(ok307)* 43, also showed an extension of DSB-1 staining to ∼90% (Figure 6B).

Significantly, mutants that lack meiotic DSBs, including *spo-11(ok79* or *me44)* 23,25, *mre-11(ok179)* 24, and *rad-50(ok197)* 25, also showed significant extension of the zone of DSB-1 staining to 69–78% of the LZP region (Figure 6A and 6B). Together these findings indicate that the absence of crossovers or crossover precursors, rather than the presence or persistence of earlier recombination intermediates, triggers extension of the DSB-1 zone.

Also of note, in *htp-1* and *htp-3* mutants 27–29, in which the axial element is disrupted, the region of DSB-1 staining was shorter than in other crossover-deficient mutants (Figure 6B), despite the fact that no crossovers form in these animals and DSBs are either eliminated or reduced 27–29. This suggests that axis structure may play a role in detecting or signaling the absence of crossover precursors to prolong DSB-1 localization, consistent with proposed roles for the axis in other species 32,69–71.

We tested whether irradiation could suppress the extension of the DSB-1 zone seen in *spo-11* mutants. Young adult hermaphrodites were irradiated, then fixed and stained 8 hours later. As controls, we included mutants (*mre-11* and *msh-5*) in which crossover defects are not rescued by exogenous DSBs 24,38. Irradiation reduced the zone of DSB-1 staining in *spo-11(me44)* animals to 56%, compared to 70% for unirradiated controls (Figure 6C). In contrast, the length of the DSB-1 zone in wild-type, *mre-11*, and *msh-5* hermaphrodites was unaffected by irradiation (Figure 6C). These data reinforce the idea that the absence of crossovers or crossover precursors induces prolonged DSB-1 association with chromosomes.

Many mutations that result in extension of the DSB-1 zone also cause elevated oocyte apoptosis, which can be triggered in response to persistent DNA damage or asynapsis 43,50,68,72. We considered the possibility that apoptosis might mediate the observed extension of DSB-1 staining, since this process primarily culls nuclei from the late pachytene, DSB-1 negative region of the gonad (reviewed in 73). To test this idea, a representative subset of meiotic mutations, including *spo-11(ok79)*, *msh-5*, *syp-2*, *him-8*, and *zim-2* (see below) were combined with *ced-4(n1162)*, which abrogates germline apoptosis 49. These double mutants displayed extended DSB-1 localization similar to that observed in the corresponding single mutants (Figure S6). We conclude that apoptosis does not account for the extension of DSB-1 staining observed in crossover-defective mutants, nor can it explain the quantitative differences observed among different mutants.

### Extension Of Dsb-1 Localization Reflects A Genome-Wide And Nucleus-Autonomous Response

To further characterize the extension of DSB-1 localization that occurs in response to defects in crossover formation, we examined mutant situations in which crossover formation was disrupted on only one chromosome. *him-8(tm611)* and *zim-2(tm574)* specifically disrupt homolog pairing and thus crossover formation on chromosomes X and V, respectively 53,74. *him-5(ok1896)* does not impair pairing or synapsis, but abrogates DSBs on the X chromosome 36. All three of these mutations extended the DSB-1 zone to 83–86% of the LZP region (Figure 6A and 6B). Furthermore, irradiation of *him-5* animals, in which the crossover defect can be rescued by exogenous DSBs 36, but not irradiation of *him-8*, suppressed the extension of DSB-1 localization (Figure 6C). These results indicate that the absence of a crossover precursor on a single chromosome pair is sufficient to prolong DSB-1 association with meiotic chromosomes.

**Figure 7:**
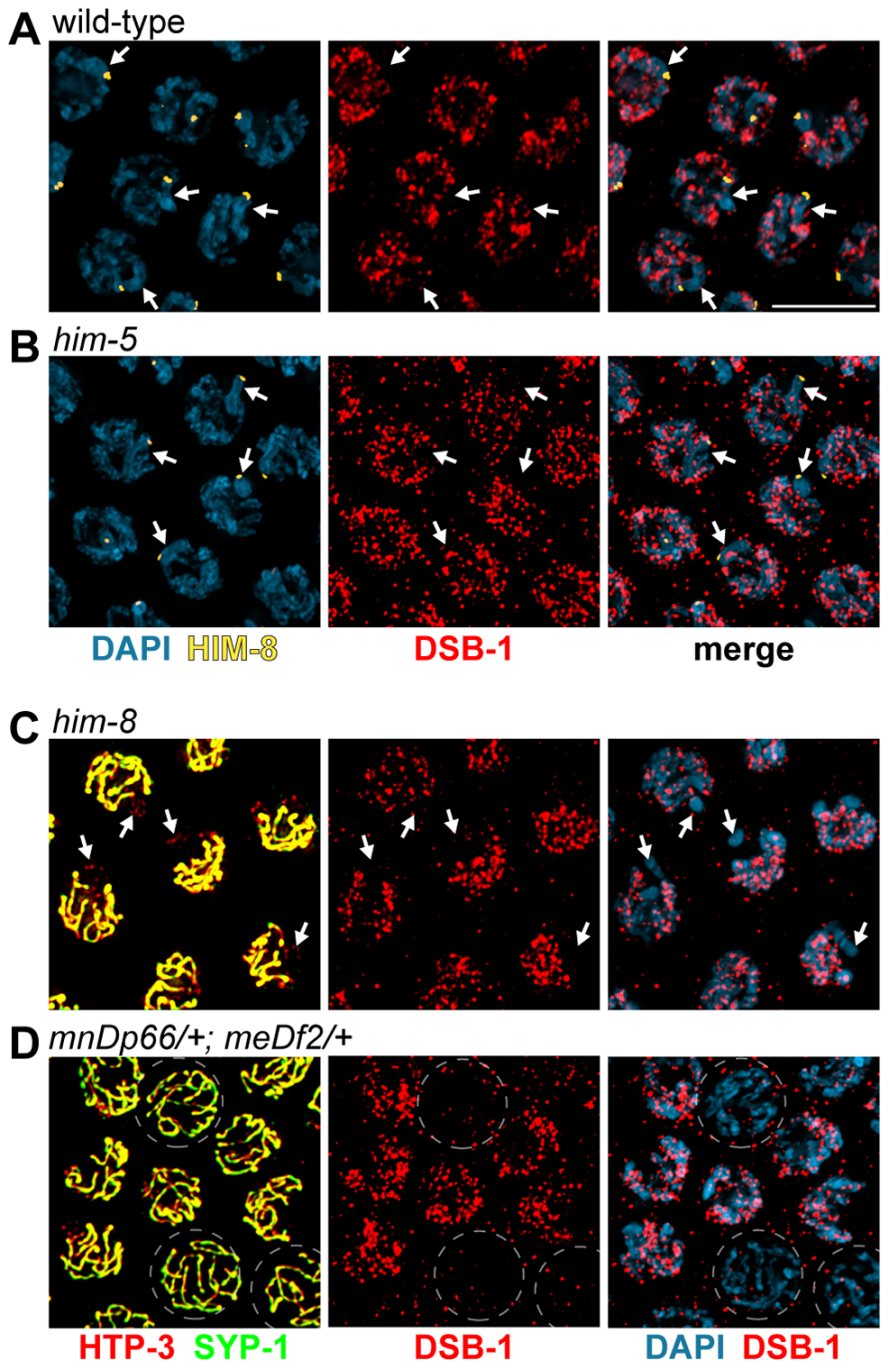
Extension of DSB-1 localization is a nuclear-autonomous response. (A, B) Immunofluorescence staining of DSB-1 and HIM-8 in pachytene nuclei in (A) WT and (B) *him-5* mutants. HIM-8 was used to mark the X chromosome (arrows). (B) Nuclei were imaged from the mid-late pachytene region of the gonad with extended DSB-1 localization. DSB-1 localization occurs throughout the nucleus and is not restricted to the X chromosome. (C) Immunofluorescence staining of DSB-1, HTP-3, and SYP-1, with DAPI in *him-8* mutants. Regions of HTP-3 staining that do not colocalize with SYP-1 identify the asynapsed X chromosomes (arrows). Nuclei from the mid-late pachytene region of the gonad, where DSB-1 would normally have disappeared, are shown. DSB-1 is observed throughout the nuclei and is not restricted to the X chromosome. (D) Hermaphrodites heterozygous for a deficiency of the X chromosome pairing center (*mnDp66/+; meDf2/+*) were stained for HTP-3, SYP-1, and DSB-1. Fully synapsed nuclei in the mid-late pachytene region lack DSB-1 staining (broken circles), while adjacent nuclei with asynapsed X chromosomes retain DSB-1 staining as well as more condensed DAPI morphology. Scale bar, 5 µm.

Analysis of mutants with chromosome-specific defects in interhomolog recombination also allowed us to test whether DSB-1 staining is specifically prolonged on crossover-deficient chromosomes. In *him-5* and *him-8* mutants, the autosomes, but not the X chromosomes, are proficient for crossover formation. X chromosomes can be specifically marked in these mutants using HIM-8 antibodies (in *him-5* mutants) or by staining for synaptonemal complex components (in *him-8* mutants). In both of these genotypes, we observed persistent DSB-1 staining on all chromosomes throughout the region of extended DSB-1 localization (Figure 7A, 7B, and 7C). As in wild-type nuclei, the X chromosome showed weaker DSB-1 staining than the autosomes (Figure 4C and 7A). These findings indicate that the extension of DSB-1 localization is a genome-wide response affecting all chromosomes within the nucleus.

To test whether the extension of DSB-1 localization is regulated by nuclear-intrinsic or extrinsic signals, we examined animals heterozygous for *meDf2*, a deficiency of the X chromosome pairing center 40. In *meDf2/+* hermaphrodites, X chromosome pairing and synapsis is partially compromised, such that approximately half the nuclei achieve full pairing and synapsis by the end of the pachytene region 75. Nuclei with asynapsed X chromosomes can be recognized by their more condensed, transition zone-like chromosome morphology, or by co-staining for axial element and central region proteins of the synaptonemal complex 75. In the late pachytene region of these animals, we found that DSB-1 staining correlated with the status of individual nuclei: those with asynapsed chromosomes were positive for DSB-1 staining, while fully synapsed nuclei lacked DSB-1 staining (Figure 7D). These results indicate that the extension of DSB-1 localization is a response to a signal intrinsic to individual nuclei, and does not extend to neighboring nuclei within the same region of the gonad. However, as in all mutants examined, DSB-1 disappeared by the end of the pachytene region of the gonad, indicative of an extrinsic, spatially regulated “override” signal that triggers progression to late pachytene and loss of the presumptive DSB-permissive state, even when crossover precursors have not been attained on all chromosomes (see Discussion).

### Functional Relationships Between Dsb-1 And Dsb-2

**Figure 8:**
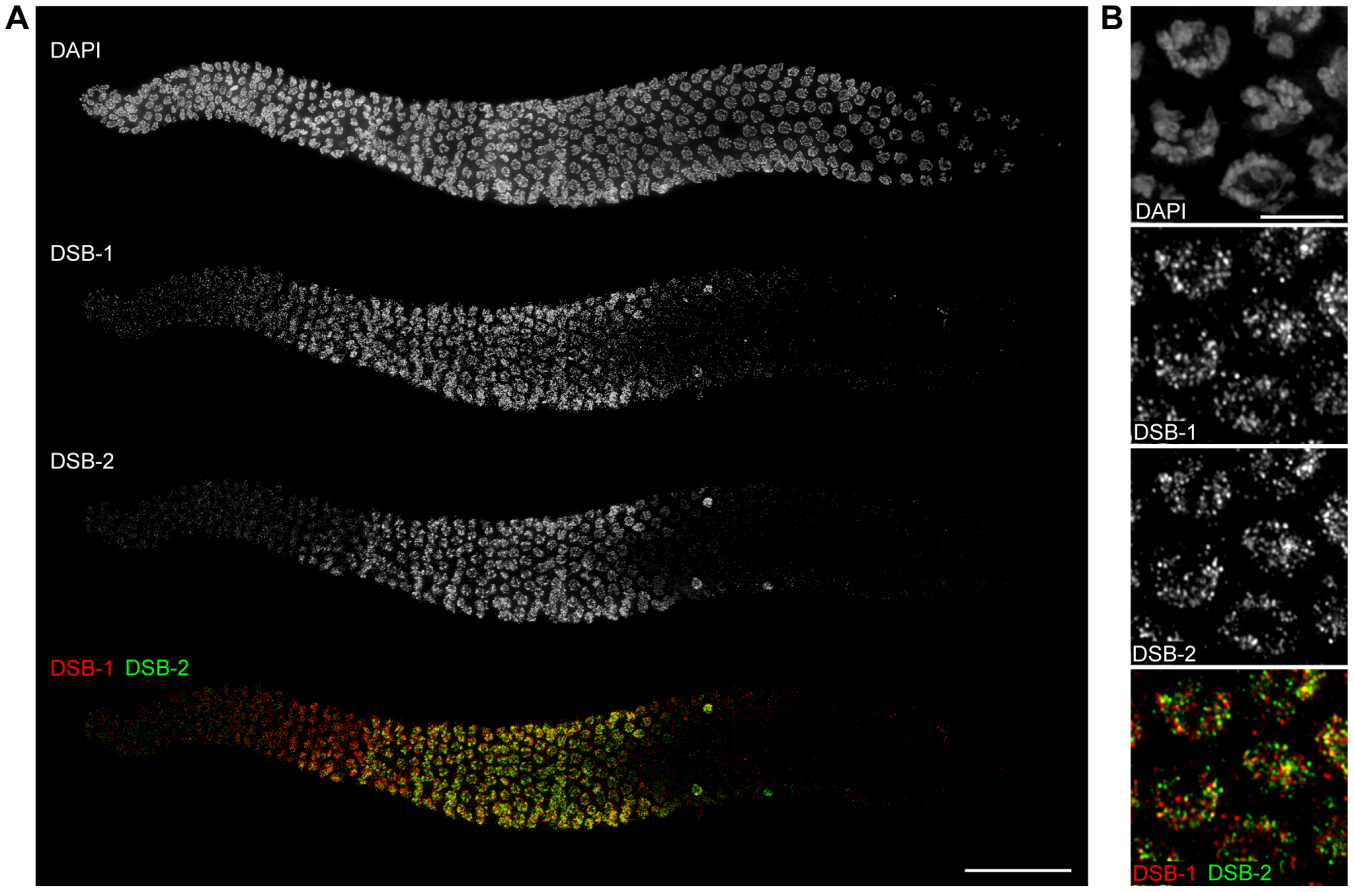
DSB-1 and DSB-2 show similar patterns of localization, but do not colocalize. (A) Composite projection image of a gonad from a WT animal showing DAPI and immunofluorescence staining for DSB-1 and DSB-2. Both proteins are detected on chromosomes by the transition zone (leptotene/zygotene), and persist until mid pachytene. Scale bar, 30 µm. (B) Projections of early pachytene nuclei showing DSB-1 and DSB-2 immunofluorescence and DAPI staining. Although DSB-1 and DSB-2 have similar staining patterns on meiotic chromosomes, they do not colocalize extensively. Scale bar, 5 µm.

The DSB-1 paralog DSB-2 is also involved in meiotic DSB formation 47. As reported in the accompanying paper by Rosu *et al.*, the two proteins show very similar localization patterns (Figure 8A and 8B, 47). Both localize to nuclei from leptotene/zygotene through mid pachytene, although DSB-1 staining appears slightly earlier than DSB-2 staining (Figure 8A). They also disappear simultaneously from meiotic chromosomes, both in wild-type animals and various mutants that disrupt crossover formation (Figure 8A, data not shown). Additionally, both proteins show similar distributions along meiotic chromosomes (Figure 8B). Intriguingly, however, the two proteins do not extensively colocalize, but instead rarely coincide (Figure 8B).

**Figure 9:**
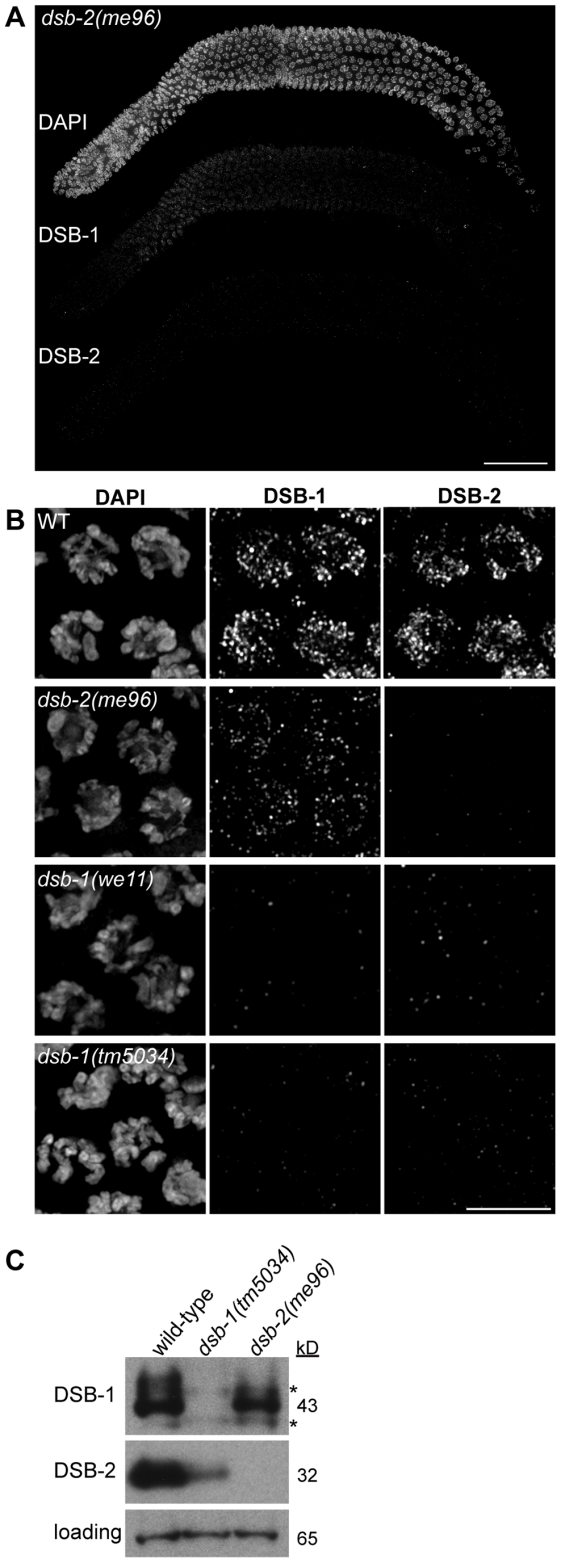
DSB-1 promotes the protein localization and protein level of DSB-2. (A) Composite projection image of a gonad from a *dsb-2(me96)* animal showing DAPI and immunofluorescence staining for DSB-1 and DSB-2. Scale bar, 30 µm. (B) Immunofluorescence staining of DSB-1 and DSB-2 in early pachytene nuclei from WT, *dsb-2(me96)*, *dsb-1(we11)*, and *dsb-1(tm5034)* animals. Reduced DSB-1 staining is observed in *dsb-2* mutants, while DSB-2 is undetectable on chromosomes in *dsb-1* mutants. Scale bar, 5 µm. (C) Immunoblotting of DSB-1 and DSB-2 in WT, *dsb-1(tm5034)*, and *dsb-2(me96)* animals. Lysate from 50 animals, 24 hours post L4, was used for each lane. DSB-1 protein levels are slightly reduced in *dsb-2* mutants, while DSB-2 protein levels are severely reduced in *dsb-1* mutants. The loading control (bottom panel) is a non-specific band. Panels were taken from the same blot. * indicates a non-specific background band.

To probe the functional interactions between DSB-1 and DSB-2, we localized each protein in the absence of the other. We found that DSB-1 localized to chromosomes in *dsb-2(me96)* mutants, although the fluorescence intensity was reduced relative to wild-type gonads (Figure 9A and 9B; see also 47). The DSB-1 positive region of the gonad was also somewhat shorter (Figure 9A), despite the reduction of crossovers in *dsb-2* mutants 47. This suggests that localization of DSB-1 to meiotic chromosomes does not require, but may be reinforced or stabilized by, DSB-2. By contrast, DSB-2 was not detected on meiotic chromosomes in *dsb-1* mutants (Figure 9B). Immunoblotting of whole-worm lysates revealed that DSB-1 protein levels are moderately reduced in *dsb-2* mutants, while DSB-2 protein levels are severely reduced in *dsb-1* mutants (Figure 9C). This parallels our conclusions from *in situ* localization of these proteins, and suggests that the reduction of staining observed on chromosomes is a consequence of lower protein levels.

**Figure 10:**
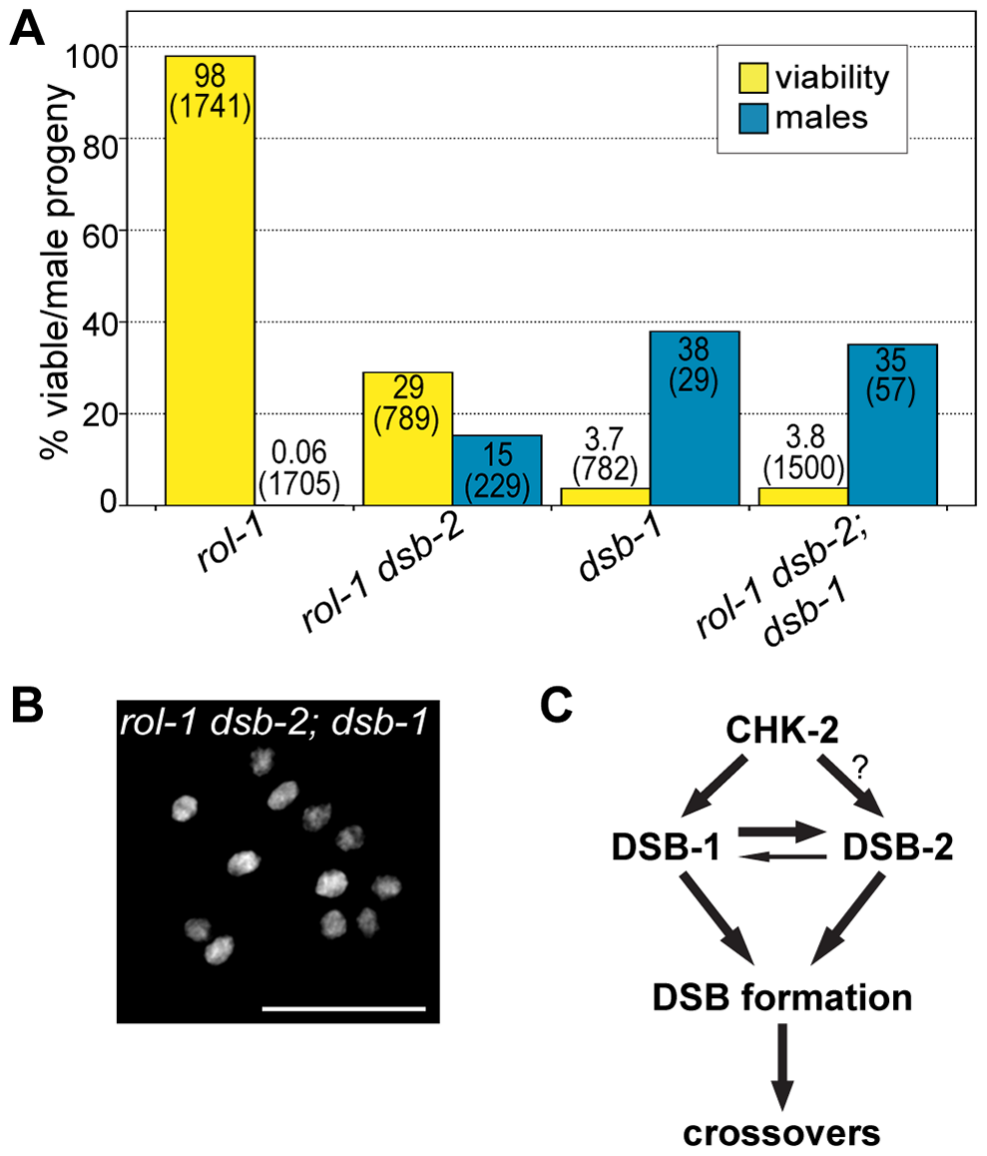
*dsb-2; dsb-1* double mutants fail to make DSBs. (A) Quantification of viable and male self-progeny produced by *rol-1*, *rol-1 dsb-2(me96)*, *dsb-1(tm5034)*, and *rol-1 dsb-2(me96); dsb-1(tm5034)* hermaphrodites. Whole broods were counted for each genotype. *dsb-2; dsb-1* double mutants display levels of progeny viability and males similar to *dsb-1* mutants. For each bar, the upper number indicates the percentage, and the lower number in parentheses indicates the total number of fertilized eggs (for viability) or adult progeny (for males) counted. (B) A representative DAPI-stained oocyte nucleus at diakinesis for *rol-1 dsb-2(me96); dsb-1(tm5034)* showing 12 DAPI-staining bodies. Scale bar, 5 µm. (C) Diagram summarizing the dependencies of DSB-1 and DSB-2 on each other and CHK-2. CHK-2 promotes the chromosomal localization of DSB-1 and DSB-2. Because DSB-2 localization depends on DSB-1, it is unknown whether CHK-2 promotes the loading of DSB-2 directly or through its effect on DSB-1. DSB-1 and DSB-2 promote or stabilize the protein levels and chromosomal localization of each other, with the effect of DSB-1 on DSB-2 being much greater than the converse. DSB-1 and DSB-2 collaborate to promote DSBs and crossover formation.

We also tested the effect of eliminating both DSB-1 and DSB-2 by constructing a double mutant strain. The phenotypes observed in *dsb-1; dsb-2* mutant animals were indistinguishable from *dsb-1* mutants (Figure 10A and 10B). This result is consistent with the idea that these proteins collaborate in some way to promote DSB formation, and argues against more complex epistasis scenarios.

## Discussion

### Dsb-1 And Dsb-2 Mediate Initiation Of Meiotic Recombination

We have discovered a novel protein, DSB-1, required for meiotic DSB formation in *C. elegans*. Our data indicate that DSB-1 acts specifically to promote DSBs, and does not play a major role in DNA repair or other meiotic processes. DSB-1 localizes to chromosomes during meiotic prophase, concomitant with the period of DSB formation. It appears more abundant on the autosomes than the X chromosome. The significance of this finding is unclear, since DSB-1 is clearly required for DSBs on all chromosomes, but it may be related to observations that the X chromosome has distinct chromatin structure and differential genetic requirements for DSB formation 35,36,51,52.

Both DSB-1 and its paralog DSB-2 are required for normal levels of meiotic DSBs. These proteins show a similar temporal and spatial pattern of localization to meiotic chromosomes. The localization of both proteins is also extended to a similar extent in mutants that disrupt crossover formation. In mutants where the localization of both DSB-1 and DSB-2 was assayed simultaneously, as well as in wild-type animals, the proteins localize to the same subset of meiotic nuclei, except that DSB-1 appears slightly earlier, suggesting that they are co-regulated. However, these proteins seem unlikely to act as a complex, since they show little if any colocalization.

Although DSB-1 and DSB-2 appear to play similar roles in meiotic DSB formation, the severity of their mutant phenotypes are not equivalent. As shown by Rosu et al., DSBs are reduced but not eliminated in young *dsb-2* mutant hermaphrodites 47, while *dsb-1* mutants lack DSBs regardless of age. The less severe defects observed in young *dsb-2* mutants likely reflect the presence of substantial residual DSB-1 protein on meiotic chromosomes in *dsb-2* mutants, whereas DSB-2 is not detected on chromosomes in *dsb-1* mutants, and protein levels are severely reduced. DSB-1 appears to stabilize DSB-2, perhaps by promoting its association with chromosomes, and to a lesser extent is reciprocally stabilized/reinforced by DSB-2.

The CHK-2 kinase promotes the chromosomal association of DSB-1. CHK-2 is also required for DSB-2 localization on meiotic chromosomes 47, although it is not clear whether CHK-2 promotes DSB-2 loading directly, or indirectly through its role in the loading of DSB-1. Our findings suggest a model in which DSB-1 and DSB-2 mutually promote each other's expression, stability, and/or localization, with DSB-2 depending more strongly on DSB-1, to promote DSB formation (Figure 10C).

The number of sites of DSB-1 and DSB-2 localization per nucleus – too many to quantify in diffraction-limited images – appears to greatly exceed the number of DSBs, estimates of which have ranged from 12 to 75 per nucleus 65,76,77. DSB-1 and DSB-2 may each bind to sites of potential DSBs, with only a subset of these sites undergoing DSB formation, perhaps where they happen to coincide. They could also be serving as scaffolds to recruit other factors required for DSB formation to meiotic chromosomes and/or to promote their functional interaction. This idea is currently difficult to test, since we have not yet been able to detect chromosomal association of SPO-11 in *C. elegans*, and no other proteins specifically required for DSBs have been identified. Alternatively, these proteins may influence DSB formation by modifying chromosome structure. We did not observe overt changes in chromosome morphology in *dsb-1* mutants, but further analysis – e.g., mapping of histone modifications – may be necessary to uncover more subtle changes.

### A Crossover Assurance Checkpoint Mechanism That Regulates Dsb Formation

DSBs normally occur within a discrete time window during early meiotic prophase. In *C. elegans* this corresponds to the transition zone and early pachytene, based on RAD-51 localization. As DSB-1 is necessary for DSB formation, and its appearance on meiotic chromosomes coincides with the timing of DSBs, we infer that the chromosomal localization of DSB-1 is indicative of a regulatory state permissive for DSB formation. We observed that when crossover formation is disrupted, this DSB-1-positive region is extended. Rosu *et al.* report a similar extension of DSB-2 in crossover-defective mutants 47.

Previous work has shown that RAD-51 foci persist longer and accumulate to greater numbers in various mutants that make breaks but not crossovers 43,64,65,67,75. Extended or elevated RAD-51 staining could reflect an extension of the time that DSBs are made, a greater number of DSBs, or a slower turnover of the RAD-51-bound state. However, persistence of DSB-1 and DSB-2 on meiotic chromosomes in these mutants suggests that the period in which nuclei are competent for DSB formation is extended. In support of this idea, in many crossover-defective mutants defective, the number of RAD-51 foci per nucleus not only reaches a far higher level but also peaks later than in wild-type animals and continues to rise even after RAD-51 is normally cleared from meiotic chromosomes 53,75,77, indicating that breaks continue to be generated after their formation would normally cease. Several mutations that impair crossover formation on a limited number of chromosome pairs result in altered crossover distributions on the crossover-competent chromosomes 64,78, which, particularly in light of the current findings, seems likely to reflect changes in DSB activity. In addition, RAD-51 chromatin immunoprecipitation data from our laboratory (C. V. Kotwaliwale and AFD, unpublished) have indicated that the DSB distribution is altered in *him-8* mutants, one of the genotypes that show extended DSB-1 staining.

Taken together, these findings strongly suggest that both the temporal and genomic distribution of DSBs is altered in many situations that perturb crossover formation. A similar phenomenon may also account for the “interchromosomal effect” first observed in *Drosophila* female meiosis 79. We note that this raises caveats about previously published estimates of DSB numbers in *C. elegans* that have been based on quantification of RAD-51 foci in genotypes that do not complete crossovers, such as *rad-54* or *syp-1* mutants 65,76,77.

Extension of the DSB-1 zone occurs even when nuclei are unable to initiate meiotic recombination due to an absence of DSBs. This suggests that the extension is not due to the persistence of unresolved recombination intermediates, but is instead a response to the absence of a particular crossover-competent recombination intermediate, or crossover precursor. We found that disruption of crossover formation on a single pair of chromosomes is sufficient to prolong DSB-1 localization on all chromosomes. Based on this result we believe that chromosomes lacking a crossover precursor may emanate a signal that sustains a DSB-permissive state within the affected nucleus. Thus, a single chromosome pair lacking a crossover precursor elicits a genome-wide response that results in extension of DSB-1 localization, which may reflect a modulation of the timing, and perhaps the extent, of DSB formation. Such a mechanism would help to ensure formation of an “obligate” crossover on every chromosome pair.

All mutants that we found to extend the localization of DSB-1 cause a disruption in crossover formation, although they have various primary molecular defects. It is possible that extension of DSB-1 localization occurs in response to distinct molecular triggers in different mutant situations. For example, *spo-11* mutants may be responding to an absence of DSBs, *rad-54* mutants to unrepaired DSBs, and *syp-1* mutants to asynapsed chromosomes. A similar model in which different unfinished meiotic tasks can elicit delays in meiotic progression was proposed in a recent study 80. However, we feel that a parsimonious interpretation of our data is that the absence of a crossover precursor on one or more chromosomes is sufficient to prolong DSB-1/2 localization. The varying degree of extension seen in different mutants could reflect the engagement of additional regulatory mechanisms, such as the synapsis checkpoint and/or DNA damage checkpoint, which might converge with a crossover assurance mechanism to modulate regulators of DSB-1.

**Figure 11:**
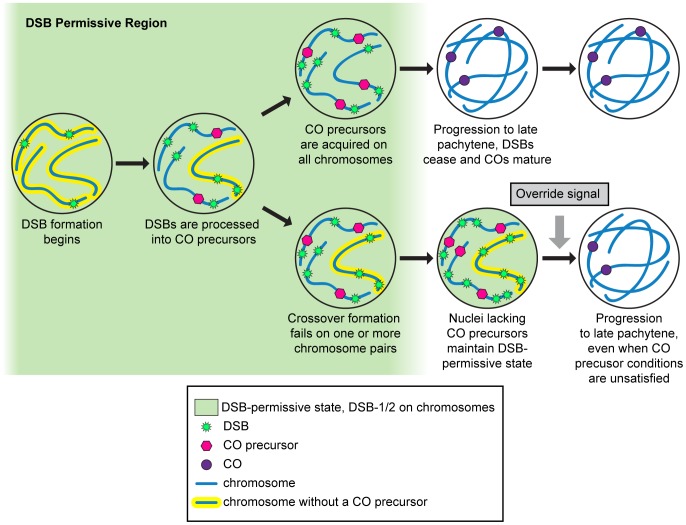
Model: Nuclei remain in a DSB-permissive state until a crossover precursor has been attained on each chromosome pair. At the onset of meiotic prophase, DSB-1 and DSB-2 are targeted to chromosomes to mediate DSB formation. A standard duration of the resulting DSB-permissive state is usually sufficient to ensure establishment of crossover (CO) precursors on most chromosome pairs. However, chromosome pairs that fail to form a crossover precursor emit a signal that prolongs the DSB-permissive state within individual nuclei. Once all chromosome pairs within a nucleus attain at least one crossover precursor, DSB-1 and DSB-2 are removed from meiotic chromosomes and DSB formation is thereby inactivated. As nuclei approach the bend region of the gonad, an override signal acts to shut off DSB formation regardless of crossover status. Upon exit from the DSB-permissive state, nuclei progress to late pachytene and complete crossover formation.

We propose that an “obligate crossover” checkpoint mediates the extension of DSB-1 localization (Figure 11). Our data suggest that DSB formation is activated during early meiosis and normally persists long enough for most nuclei to attain crossover precursors on all chromosomes (Figure 11). If interhomolog recombination is impaired on one or more chromosome pairs, individual nuclei can prolong the DSB-permissive state in an attempt to generate a crossover on every chromosome. Our observation that a block to crossover formation on a single pair of chromosomes results in persistent DSB-1 throughout the affected nuclei is reminiscent of the spindle assembly checkpoint (SAC), in which failure of a single pair of sister kinetochores to biorient on the mitotic spindle triggers a cell-autonomous delay in anaphase onset that affects cohesion on all chromosomes 81. Interestingly, a key mediator of the SAC, Mad2, is homologous to the meiotic axis proteins HTP-3 and HTP-1 28,82, which appear be important for the regulatory circuit that mediates prolonged DSB-1 localization in response to crossover defects.

An alternative model would be a negative feedback circuit in which the acquisition of all necessary crossover-intermediates triggers inactivation of DSB formation. According to this view, the presence of crossover precursors generates a signal to exit the DSB permissive state, rather than the absence of precursors extending this period. Such a model would require a ‘counting’ mechanism that enables exit from the DSB permissive state in response to a threshold number of crossover precursors. This seems less likely based on first principles, and also less consistent with our data.

Our observations also suggest that there is a minimum duration of proficiency for DSB formation that does not depend on how rapidly chromosome pairs attain crossover precursors. We would expect meiotic nuclei to achieve crossover precursors on every chromosome in a stochastic manner. If DSB-1 were removed from chromosomes upon reaching this state, we would likely see a patchwork of DSB-1 positive and negative nuclei in the early pachytene region, but instead we observe homogenous staining in this region, and abrupt disappearance of DSB-1 within a narrow zone of the gonad. Additionally, in mutants that appear to be defective in triggering the obligate crossover checkpoint, such as *htp-3* and *htp-1*, a zone of DSB-1-positive nuclei similar in length to that in wild-type animals is observed. Together these observations suggest that there is a preset temporal window for DSB formation that can be extended in individual nuclei but not shortened.

The duration of the DSB-permissive state might be specified by an activity or signal that decays with time and/or distance after meiotic entry. We speculate that the disappearance of DSB-1 may reflect a drop below a threshold level of CHK-2 activity, decay of CHK-2-mediated phosphorylation of DSB-1 or other targets, and/or a rise in an opposing activity – *e.g.*, a phosphatase. Any of these could be inhibited by the putative checkpoint mechanism that prolongs DSB-1 localization in response to impaired crossover formation.

The nature of the recombination intermediate that satisfies the requirement for a crossover precursor on all chromosomes remains unknown. We distinguish “crossover precursors” from “interhomolog recombination intermediates” because components that are specifically required for crossovers, including MSH-5, ZHP-3, and COSA-1 38,39,66,83, are all required for timely disappearance of DSB-1 from chromosomes. However, cytological markers for crossovers, including foci of ZHP-3 and COSA-1, do not appear until the late pachytene region of the gonad 39,66, after DSB-1 and DSB-2 disappear from meiotic chromosomes 47. Thus, it seems likely that crossover precursors, rather than mature crossovers, are sufficient to allow exit from the DSB-permissive state.

Genetic and cytological evidence indicate that nuclei eventually cease to make DSBs, even when crossovers fail to be made on one or more chromosomes. As nuclei approach the bend region of the gonad at the end of pachytene, an “override” signal appears to shut off DSB formation (Figure 11). Unlike in mammals, where crossover failures result in extensive apoptosis 84, *C. elegans* hermaphrodites produce both sperm and oocytes in roughly normal numbers even when homolog pairing, synapsis, and/or recombination are severely impaired.

### The Relationship Between The Crossover Assurance Mechanism And Meiotic Progression

Numerous studies have documented a phenomenon known as the “extended transition zone” in mutants with defects in homolog pairing and/or synapsis 30,43,53,68. An extended transition zone has been defined as a longer region of the gonad containing nuclei with crescent-shaped DAPI-staining morphology, multiple patches of the nuclear envelope proteins SUN-1 and ZYG-12, and strong foci of the ZIM proteins 68,74,85. An extended transition zone appears to be a response to asynapsed chromosomes 43,68. Previous work from our lab showed that the extension of the transition zone in synapsis-defective animals such as *him-8* hermaphrodites was suppressed by mutations in recombination factors, including *spo-11* and *msh-5*, and we therefore proposed that it might reflect a response to unresolved recombination intermediates 64. However, subsequent work has revealed that these double mutant situations actually resulted in precocious fold-back synapsis of unpaired chromosomes, thereby silencing the asynapsed chromosome response (SER and AFD, unpublished).

Since mutations that abrogate pairing or synapsis also impair interhomolog recombination, it is not surprising that most genotypes with extended transition zones also show persistent DSB-1 localization. However, not all mutants that disrupt crossover formation extend the transition zone. *spo-11 and him-5* mutants, for example, are deficient for DSB formation on one or more chromosomes and show extended DSB-1 staining, but do not show typical extended transition zones. Instead, these mutants appear to have extended regions of early pachytene nuclei. Based on these observations, we believe that the obligate crossover checkpoint mechanism is distinct from the response to asynapsed chromosomes. However, these two regulatory circuits serve similar purposes – to enable meiotic nuclei more time to complete synapsis or achieve crossovers on all chromosomes – and they may also involve common molecular components.

### Rapid Divergence Among Dsb-Promoting Proteins

Proteins with apparent homology to DSB-1 are restricted to the *Caenorhabditis* lineage. Even within Caenorhabditids, DSB-1, DSB-2 and their homologs are only weakly conserved. This reinforces abundant evidence from other organisms that apart from Spo11 itself and the Rad50-Mre11 complex, proteins that promote DSB formation diverge rapidly during evolution 17,18,86. This might seem surprising given that meiotic DSB formation is an essential aspect of sexual reproduction in most eukaryotes. However, potent and acute evolutionary pressures act on meiosis. For example, the germline is the site of intense warfare between the host genome and selfish genetic elements, which may contribute to the rapid evolution of meiotic proteins. In addition, the genome-wide distribution of DSBs appears to underlie the strongly biased distribution of crossovers observed in many species 87,88, including *C. elegans* (C. V. Kotwaliwale and AFD, unpublished). The nature of this biased distribution shows interesting variation among species 89,90. Since crossover number and position have a direct impact on the fidelity of meiotic chromosome segregation, mechanisms governing DSB distribution have likely evolved in concert with changes in chromosome structure and the spindle apparatus to maintain reproductive fitness.

Several features of meiosis in *C. elegans* distinguish it from other organisms in which DSB-promoting factors have been identified. In particular, DSBs and early recombination steps contribute directly to homolog pairing and synapsis in many species, while in *C. elegans* homolog pairing and synapsis occur independently of DSBs. Additionally, *C. elegans* lacks Dmc1, Hop2, and Mnd1, which are thought to function together as an essential meiotic recombination module in most eukaryotes 91. *C. elegans* also lacks the DSB proteins Mei4 and Rec114, which are conserved between budding yeast and mice 18. A correlation between the absence of DMC1/Hop2/Mnd1 and Mei4/Rec114 has been noted in several other lineages, and has been suggested to reflect a functional link between the formation of DSBs and their subsequent repair 18. Interestingly, Rec114, like DSB-1/2, has several potential target sites for ATM/ATR phosphorylation, and these are important for regulation of DSBs in budding yeast meiosis 14. Thus, the DSB-1/2 family of proteins may play analogous roles to known mediators of DSB formation in other species, despite their lack of apparent sequence similarity.

## Materials And Methods

### *C. Elegans* Mutations And Strains

All *C. elegans* strains were cultured under standard conditions at 20°C. The wild-type strain was N2 Bristol. The nonsense *dsb-1(we11)* allele was generated by EMS mutagenesis. The *dsb-1* deletion allele *(tm5034)* was generated by the Japanese National BioResource for the Nematode. Both *dsb-1* alleles were extensively outcrossed to wild-type (5-6x), and additionally outcrossed in a directed three-point cross to *dpy-20 unc-30* to eliminate most linked mutations. Additional mutants analyzed in this study were: *spo-11(me44, ok79)*, *mre-11(ok179)*, *rad-50(ok197)*, *chk-2(me64)*, *com-1(t1626)*, *rad-51(Ig8701)*, *rad-54(tm1268)*, *msh-5(me23)*, *cosa-1(me13)*, *zhp-3(jf61)*, *htp-3(tm3655)*, *htp-1(gk174)*, *syp-1(me17)*, *syp-2(ok307)*, *him-8(tm611)*, *zim-2(tm574)*, *dsb-2(me96)*, and *ced-4(n1162)*. Strains used in this study were:

- CA1104 *dsb-1(we11) IV/nT1[unc-?(n754) let-?] (IV;V)*
- CA1105 *dsb-1(tm5034) IV/nT1[unc-?(n754) let-?] (IV;V)*
- CA279 *spo-11(me44)/mIs11 IV*
- CA276 *spo-11(ok79)/mIs11 IV*
- CA1109 *mre-11(ok179) V/nT1[unc-?(n754) let-?] (IV;V)*
- AV158 *rad-50(ok197) V/nT1[unc-?(n754) let-? qIs50] (IV;V)*
- CA1110 *chk-2(me64) rol-9(sc148)/+ V*
- GE4132 *unc-32(e189) com-1(t1626)/qC1 dpy-19(e1259) glp-1(q399) III*
- CA538 *rad-51(Ig8701)/mIs11 IV*
- CA855 *rad-54(tm1268) I/hT2[bli-4(e937) let-?(q782) qIs48] (I;III)*
- AV115 *msh-5(me23) IV/nT1[unc-?(n754) let-?] (IV;V)*
- AV424 *cosa-1(me13)/qC1[dpy-19(e1259) glp-1(q339) qIs26] III*
- CA685 *zhp-3(jf61) I/hT2[bli-4(e937) let-?(q782) qIs48] (I;III)*
- CA821 *htp-3(tm3655) I/hT2[bli-4(e937) let-?(q782) qIs48] (I;III)*
- AV393 *htp-1(gk174) IV/nT1[unc-?(n754) let-?] (IV;V)*
- AV307 *syp-1(me17) V/nT1[unc-?(n754) let-? qIs50] (IV;V)*
- AV276 *syp-2(ok307) V/nT1[unc-?(n754) let-?] (IV;V)*
- CA257 *him-8(tm611) IV*
- CA258 *zim-2(tm574) IV*
- AV477 *dsb-2(me96) II*
- AV539 *rol-1(e91) dsb-2(me96)/mnC1[dpy-10(e128) unc-52(e444)] II*
- CA1111 *rol-1(e91) dsb-2(me96)/mnC1[dpy-10(e128) unc-52(e444)] II; dsb-1(tm5034)/mIs11[let-?] IV*
- CB91 *rol-1(e91) II*
- CA1087 *ced-4(n1162) III*
- CA1090 *ced-4(n1162) III; spo-11(ok79)/mIs11 IV*
- CA1092 *ced-4(n1162) III; msh-5(ne23) IV/nT1[unc-?(n754) let-?] (IV;V)*
- CA1091 *ced-4(n1162) III; syp-2(ok307) V/nT1[unc-?(n754) let-?] (IV;V)*
- CA1088 *ced-4(n1162) III; him-8(tm611) IV*
- CA1989 *ced-4(n1162) III; zim-2 (tm574) IV*
- DP38 *unc-119(ed3) III*

### Quantification Of Viability And Male Progeny

L4 hermaphrodites were picked onto individual plates and transferred to new plates every 12 hours, for a total of 6–8 12-hour laying periods, until newly-laid fertilized eggs were no longer observed. Eggs were counted immediately after each 12-hour laying period. Surviving hermaphrodite and male progeny were counted 3 days later.

### Immunofluorescence And Cytological Analysis

Polyclonal antibodies against recombinant full-length DSB-1 protein were produced at Pocono Rabbit Farm & Laboratory. 6xHis-DSB-1 was purified from *E. coli* using Ni beads under denaturing conditions. The protein was resolved on an SDS-PAGE gel and the excised DSB-1 band was used to immunize guinea pigs. Rabbit anti-HTP-3 antibodies were raised against a synthetic peptide (PTEPASPVESPVKEQPQKAPK) by Strategic Diagnostics Inc., SDIX. Additional antibodies used in this study were: guinea pig anti-HTP-3 75, rat anti-HIM-8 53, rabbit anti-RAD-51 92, goat anti-SYP-1 92, and rabbit anti-DSB-2 47.

Immunofluorescence was performed as previously described 93. Briefly, hermaphrodites 24–28 hours post L4 were dissected in egg buffer containing sodium azide and 0.1% Tween 20, fixed for 3 min in 1% formaldehyde in the same buffer between a Histobond slide and coverslip, and frozen on dry ice. The coverslip was removed, and slides were transferred to methanol chilled to −20°C. After 1 min, slides were transferred to PBST (PBS containing 0.1% Tween 20), washed in two further changes of PBST, blocked with Roche blocking agent, and stained with primary antibodies in block for 2 hours at room temperature or overnight at 4°C. Slides were then washed with 3 changes of PBST and stained with secondary antibodies. Secondary antibodies labeled with Alexa 488, Cy3, or Cy5 were purchased from Invitrogen or Jackson ImmunoResearch. Following immunostaining, slides were washed, stained in 0.5 mg/ml DAPI, destained in PBST, and mounted in buffered glycerol-based mounting medium containing 4% n-propyl gallate as an antifading agent.

For quantification of DAPI-staining bodies in oocytes, animals were dissected, fixed, and DAPI-stained as described above, omitting the steps involving immunostaining.

FISH procedures have also been previously described in detail 93. Probes used in this study included the 5S rDNA repeat 23 and a short repeat associated with the right end of the X chromosome 53.

All images were acquired using a DeltaVision RT microscope (Applied Precision) equipped with a 100× 1.40 oil-immersion objective (Olympus) or (for whole gonad images) a 60× 1.40 oil-immersion objective (Olympus). Image deconvolution and projections were performed with the softWoRx software package (Applied Precision). Image scaling, false coloring, and composite image assembly were performed with Adobe Photoshop. All micrographs presented in the figures are maximum-intensity projections of 3D data stacks.

### Immunoblotting

Lysate from 50 young adult hermaphrodites, picked at 24 hours post L4, was used for each lane. Gel electrophoresis was performed using 4–12% Novex NuPage gels (Invitrogen). Proteins were transferred to PVDF membrane. Guinea pig DSB-1 antibodies and rabbit DSB-2 antibodies (see above) were used for immunoblotting, followed by detection with HRP-conjugated secondary antibodies and ECL Western Blotting Substrate (Pierce).

### Irradiation Experiments

Young adult worms were irradiated with approximately 10 Gy (1000 rad) from a Cs-137 source. For each experiment, unirradiated controls were treated identically to irradiated animals, other than exposure to radiation. For quantification of DAPI-staining bodies at diakinesis, hermaphrodites were irradiated 4–5 hours post L4 and dissected 18 hours post irradiation. To assess progeny survival, animals were irradiated 4–5 hours post L4, eggs laid 20–30 hours post irradiation were quantified, and surviving progeny were quantified 3 days later. For quantification of DSB-1 localization, animals were irradiated 16 hours post L4 and dissected 8 hours post irradiation. For RAD-51 immunofluorescence, animals were irradiated 24 hours post L4 and dissected 1 hour post irradiation.

### Whole Genome Sequencing Of *We11*

1000 homozygous *we11* animals were picked from an outcrossed, balanced strain. A genomic DNA library was prepared as described in the genomic DNA library protocol from Illumina. Libraries were sequenced using 76-bp single-end Illumina sequencing. MAQGene 94 was used to identify mutations present in the *we11* mutant strain.

### Germline Cosuppression

A 2.1-kb region of genomic DNA including the *dsb-1* coding sequence and promoter was amplified by PCR using the following primers: 5′-CCGCTTCCGAATACCGCC-3′ and 5′-GGTGCCGCTGTGTAGAAGAAGC-3′. 100 ng/µl of *dsb-1* PCR product was combined with 50 ng/µl of unc-119 rescuing plasmid pMM051 95 and injected into *unc-119* animals. Rescued non-Unc F1 animals were picked to individual plates and assayed for embryonic lethality and male progeny. F2 animals were dissected, stained, and observed to quantify the number of DAPI-staining bodies in oocytes at diakinesis.

### Quantitative Rt-Pcr

12 young adult animals, 24 hours post L4, were used for each genotype. RNA was purified from animals and reverse transcribed into cDNA with the SuperScript kit from Invitrogen using poly-A primers. *spo-11* mRNA levels were compared by real-time PCR analysis with SYBR Green (Kapa Biosystems). *act-1* and *htp-3* mRNA levels were used as normalization controls. Primers used were as follows: *spo-11* (5′-TGAGCCCGGATCTGTAGAAT-3′, 5′-TAGCTTGTTCCTTCGGTGGT-3′), *act-1* (5′-CCCCATCAACCATGAAGATC-3′, 5′-TCTGTTGGAAGGTGGAGAGG-3′), and *htp-3* (5′-CGAGTGATGACAGGGCTATATTC-3′, 5′-TGCAAGATAAACGCAGTTGG-3′).

## Supporting Information

Figure S1 Mutation of *dsb-1* does not affect *spo-11* expression. Real-time PCR was used to measure the levels of *spo-11* mRNA in *dsb-1* mutants and WT animals. RNA was purified from age-matched young adult hermaphrodites at 24 hours post-L4. *spo-11* mRNA levels were normalized either to (A) *act-1* or (B) *htp-3* mRNA levels, both of which gave similar results. (TIF)

Figure S2 Amino acid alignment of DSB-1 homologs. Global alignment of DSB-1 homologs from *C. elegans*, *C. briggsae*, *C. remanei*, and *C. japonica*. Two genes with homology to DSB-1 and DSB-2 were identified in the genome of each species included here. Alignment was performed using Geneious Pro (Geneious alignment, Blosum62, default settings). (TIF)

Figure S3 Validation of DSB-1 antibody specificity. Immunofluorescence staining of DSB-1 in early pachytene nuclei in *dsb-1* mutants. Only faint nonspecific background staining is observed. Scale bar, 5 µm. (TIF)

Figure S4 DSB-1 positive nuclei in the late pachytene region display RAD-51 foci and regions of asynapsed chromosomes. (A) Immunofluorescence staining of DSB-1 and RAD-51 in nuclei from the late pachytene region of the gonad. Nuclei positive for DSB-1 staining also show condensed, transition zone-like DAPI-staining morphology, and have abundant RAD-51 foci. (B) Immunofluorescence staining of DSB-1, HTP-3, and SYP-1 in nuclei from the late pachytene region of the gonad. Nuclei positive for DSB-1 staining contain asynapsed chromosome regions (HTP-3 positive axes not associated with SYP-1). DSB-1 positive nuclei are outlined with a dotted line. (TIF)

Figure S5 Extension of DSB-1 staining is correlated with the extension of RAD-51 staining in mutants that disrupt crossover formation. Composite projection image of a gonad from a *him-8* hermaphrodite, showing DAPI and immunofluorescence staining for DSB-1 and RAD-51. The disappearance of DSB-1 coincides with the disappearance of RAD-51 foci. (TIF)

Figure S6 Extension of the DSB-1 region in crossover-deficient mutants is not a consequence of apoptosis. Quantification of the zone of DSB-1 localization, showing the percent, by length, of the LZP region positive for DSB-1 staining. The genotypes indicated along the x-axis are present either as single mutants in the wild-type *ced-4* background or as double mutants combined with *ced-4(n1162)*. Mutation of *ced-4* abrogates germline apoptosis, but does not markedly or consistently alter the extended zone of DSB-1 localization to chromosomes. Error bars indicate standard deviations. (TIF)
