# Correction: Identification of DSB-1, a Protein Required for Initiation of Meiotic Recombination in *Caenorhabditis elegans*, Illuminates a Crossover Assurance Checkpoint

**DOI:** 10.1371/annotation/340dc359-b1f8-46bb-a82d-d209315c154c

**Published:** 2013-09-23

**Authors:** Ericca L. Stamper, Stacia E. Rodenbusch, Simona Rosu, Julie Ahringer, Anne M. Villeneuve, Abby F. Dernburg

In some subheadings and in the body of the text there are errors in the capitalization of specific terms. Please see the following list detailing these changes:

Dsb should appear as DSB

Dsb-1 should appear as DSB-1

Dsb-2 should appear as DSB-2

Spo-11 should appear as SPO-11

Mrn should appear as MRN

Chk-2 should appear as CHK-2

Rt-Pcr should appear as PT PCR

These errors appear in the HTML version of the article but not the PDF, with the exception of the following, which appears in both versions:

“A correlation between the absence of DMC1/Hop2/Mnd1 and Mei4/Rec114 has…” should appear as “A correlation between the absence of Dmc1/Hop2/Mnd1 and Mei4/Rec114 has…”

