# Supplementary figures and images for: Identification of DSB-1, a Protein Required for Initiation of Meiotic Recombination in Caenorhabditis elegans, Illuminates a Crossover Assurance Checkpoint
Source: PLoS Genet. 2013 Aug 8;9(8):e1003679. doi: 10.1371/journal.pgen.1003679 (PMC3749324; doi:10.1371/journal.pgen.1003679)

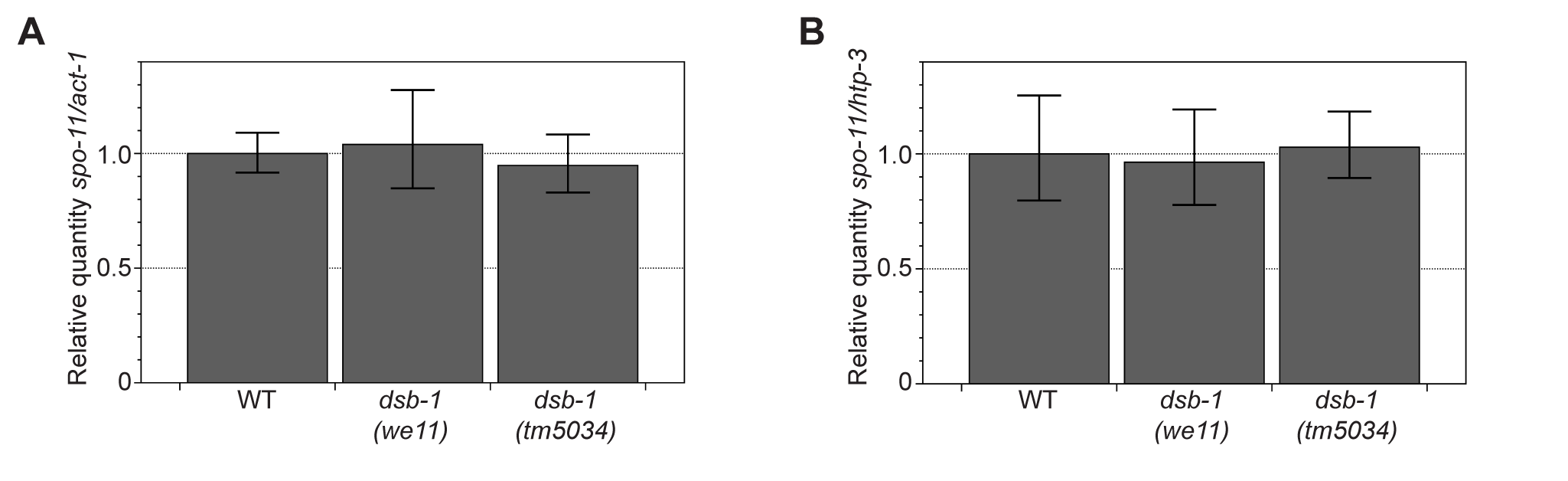

Supplement: Figure S1 — (TIF) [file pgen.1003679.s001.tif]

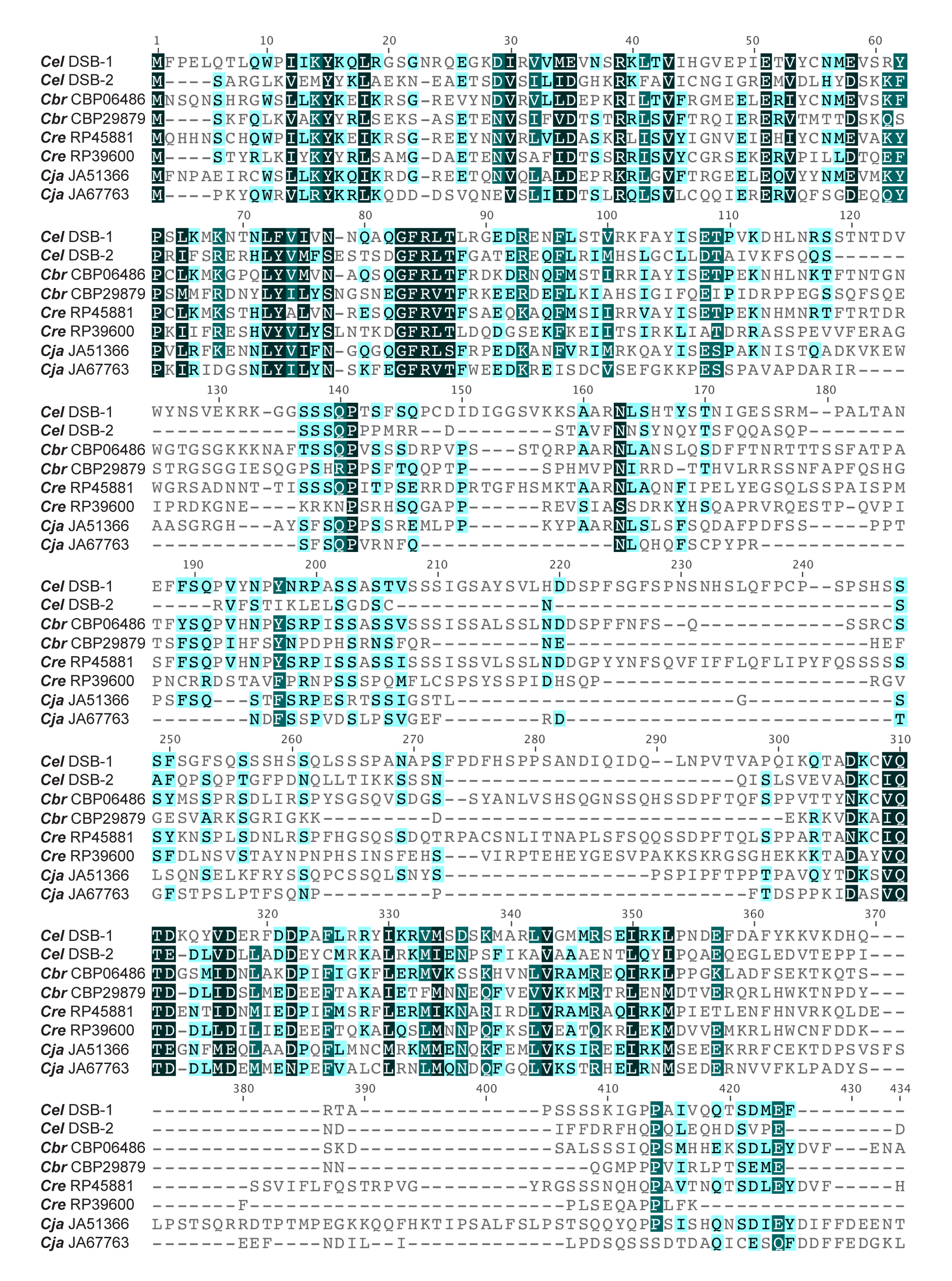

Supplement: Figure S2 — (TIF) [file pgen.1003679.s002.tif]

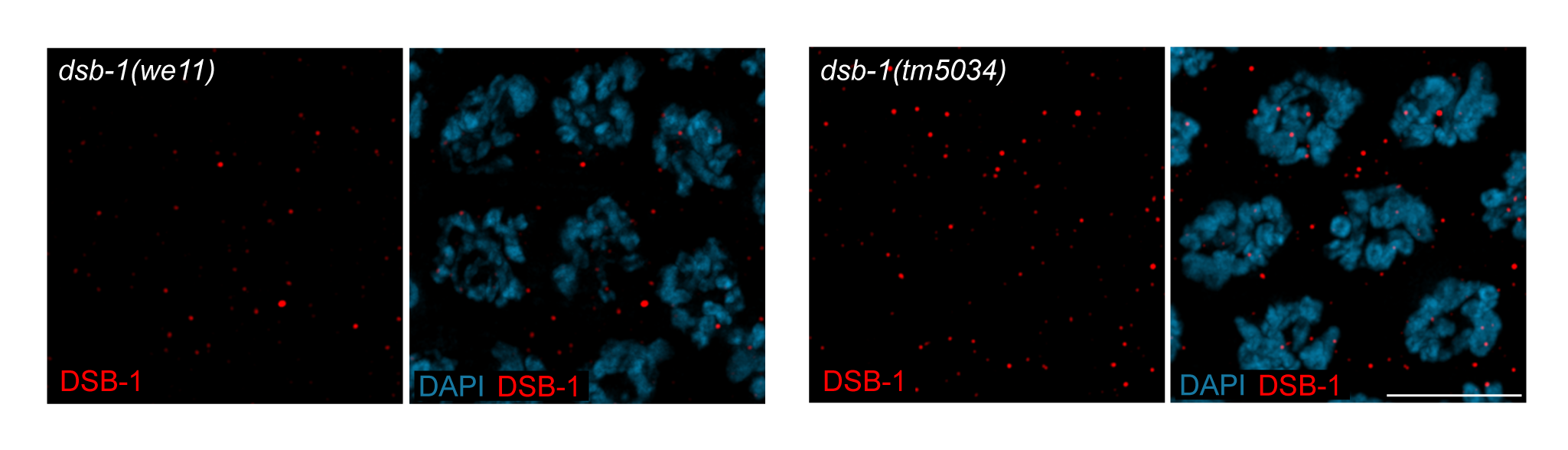

Supplement: Figure S3 — (TIF) [file pgen.1003679.s003.tif]

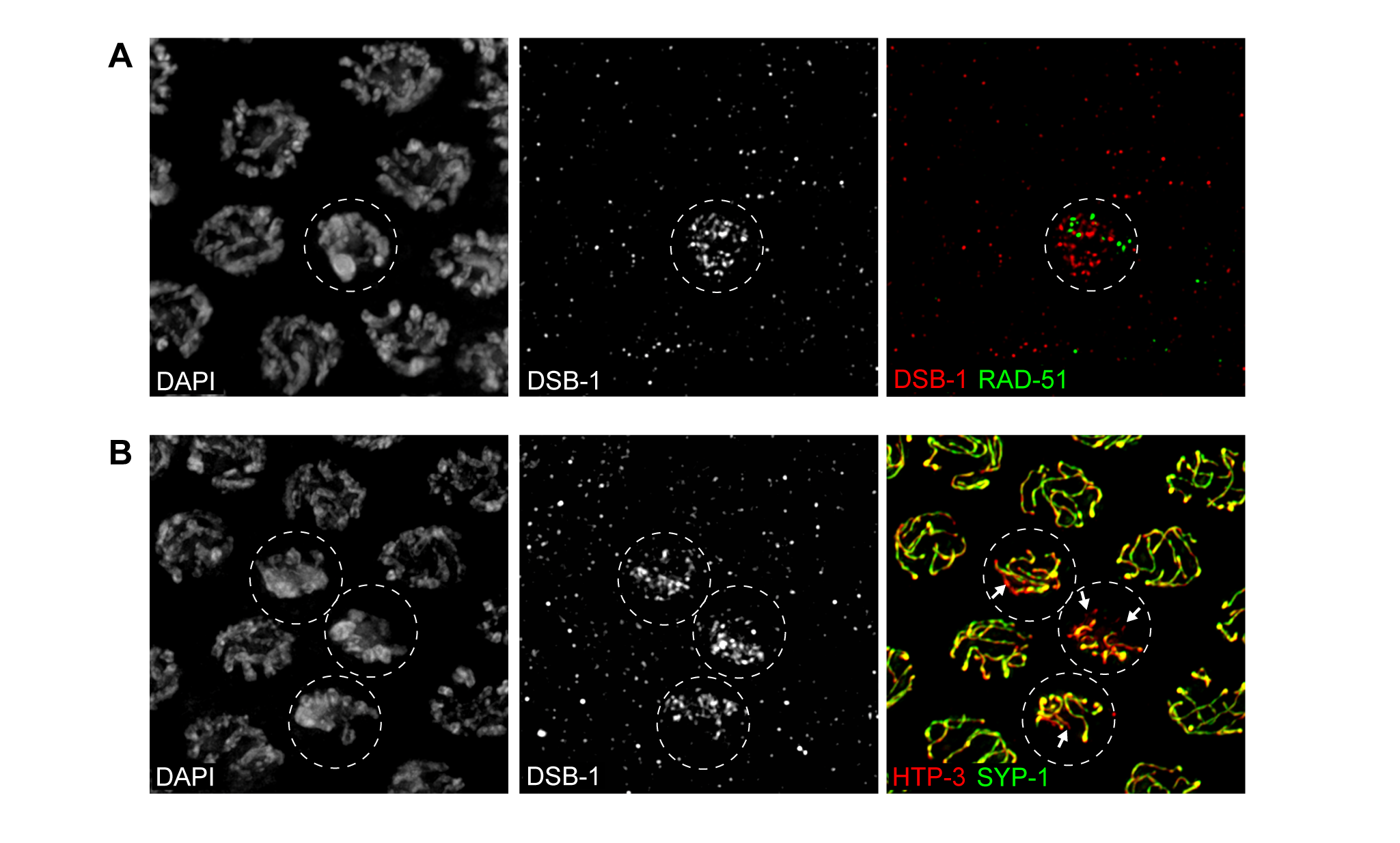

Supplement: Figure S4 — (TIF) [file pgen.1003679.s004.tif]

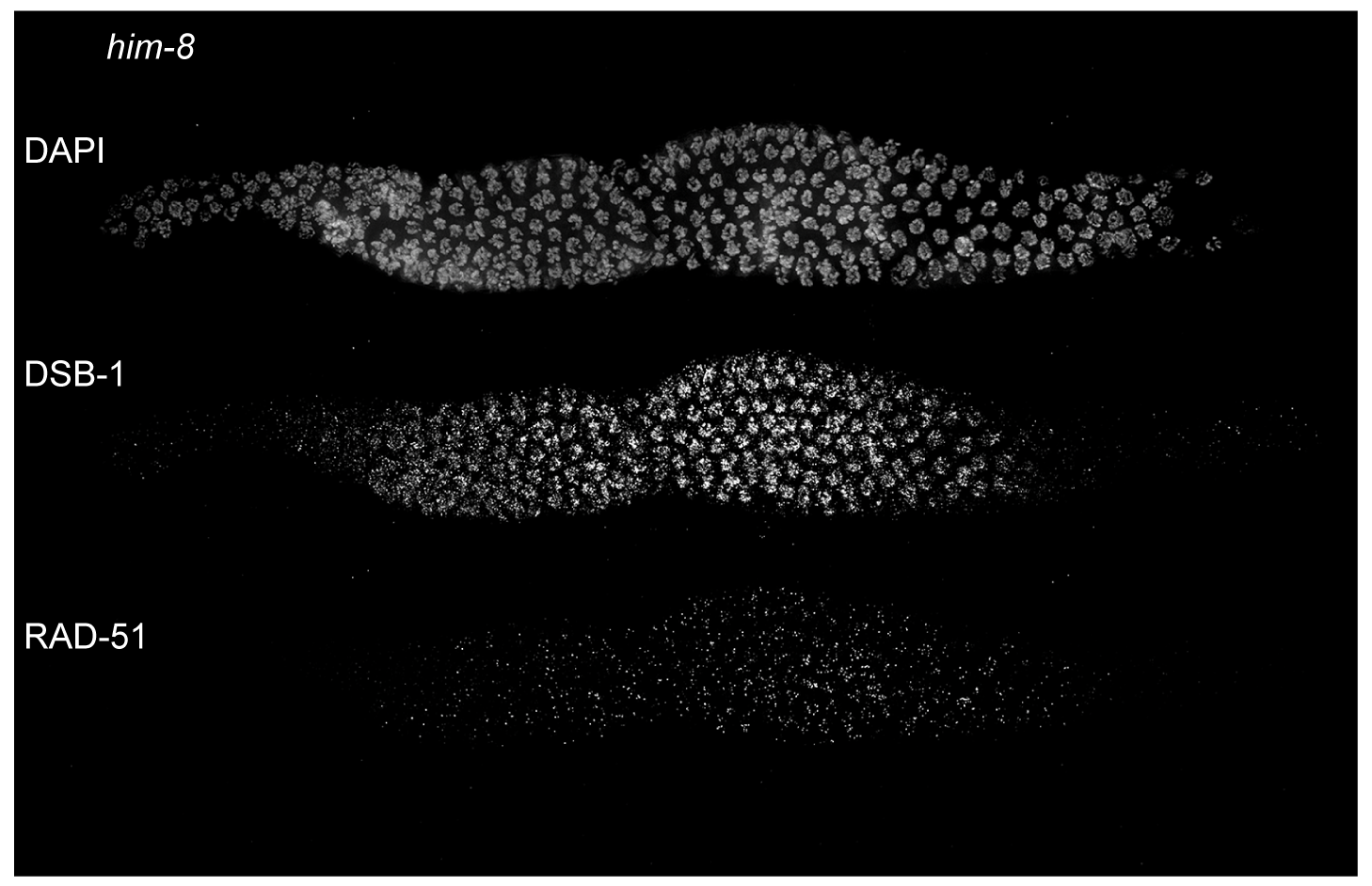

Supplement: Figure S5 — (TIF) [file pgen.1003679.s005.tif]

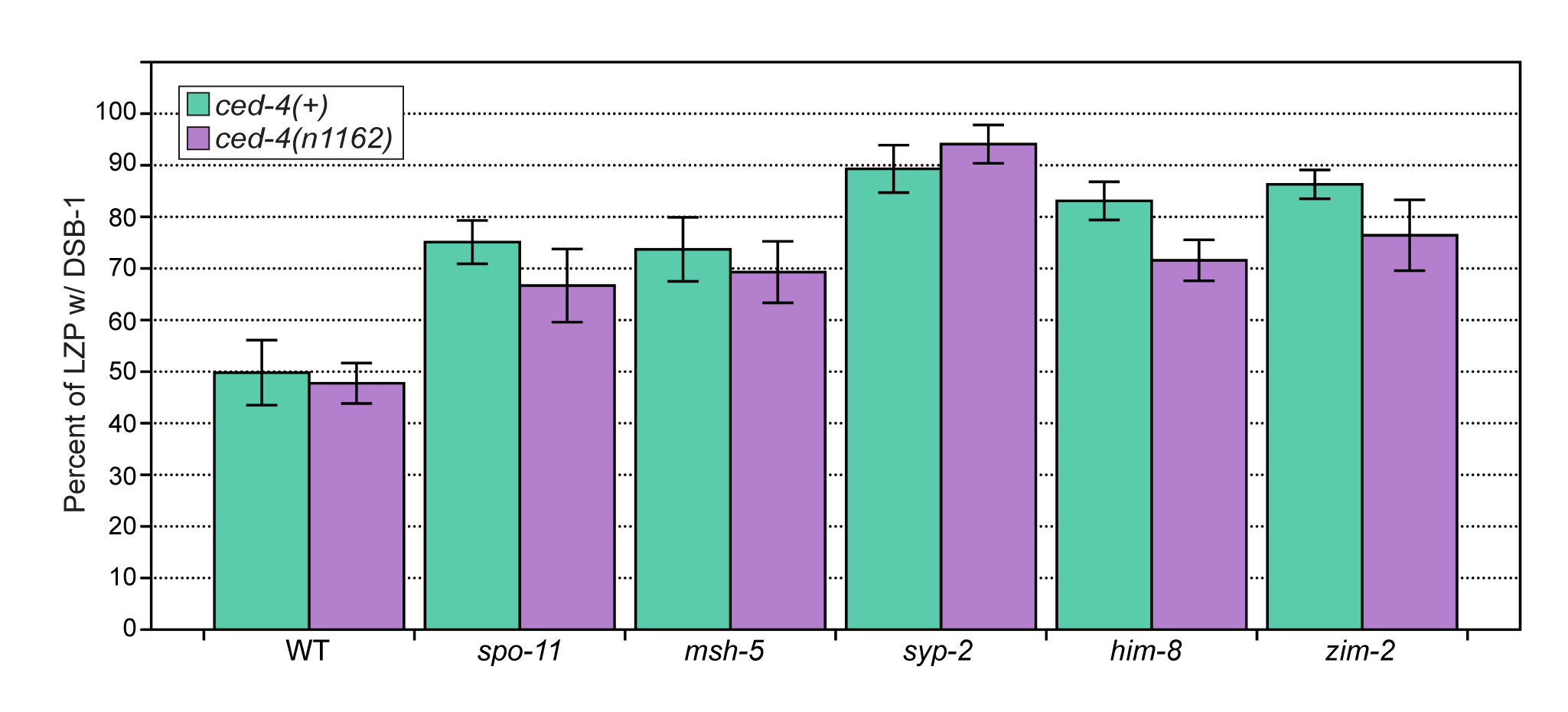

Supplement: Figure S6 — (TIF) [file pgen.1003679.s006.tif]
